# Virtual-AMR LiDAR Fusion for AMCL Localization Under Mutual Occlusion

**DOI:** 10.3390/s26196184

**Published:** 2026-09-29

**Authors:** Chin-Sheng Chen, Chia-Jen Lin, Yuan-Chih Lee, Feng-Chieh Lin

**Affiliations:** 1 Graduate Institute of Automation Technology, National Taipei University of Technology, Taipei 10608, Taiwan; saint@ntut.edu.tw; 2 Department of Electrical Engineering, National Yunlin University of Science and Technology, Yunlin 64002, Taiwan; cjlin@yuntech.edu.tw; 3 AUO Corporation, No. 1, Li-Hsin Rd. 2, Hsinchu Science Park, Hsinchu City 30078, Taiwan; yanchihlee@auo.com

**Keywords:** autonomous mobile robot, adaptive Monte Carlo localization, mutual occlusion, LiDAR fusion

## Abstract

When autonomous mobile robots (AMRs) travel in formation, mutual occlusion reduces the map features available to adaptive Monte Carlo localization (AMCL) and introduces robot body returns. This study proposes a Virtual-AMR LiDAR fusion method that transforms synchronized 2D scans into a common frame, tracks robot contours, estimates relative pose by KD-tree overlap matching, removes robot returns, and fuses environmental measurements for AMCL. The method was evaluated using two AMRs in a 3.1 m × 10 m indoor area with 4 m straight-line and turning trajectories. Four branch-wise configurations were tested in five repeated trials. Against an internal reference trajectory constructed from the planned path and motor encoder measurements rather than an independent ground truth system, the complete method achieved trial-level 2D RMSE values of 0.0412 ± 0.0083 m for the straight-line trajectory and 0.0511 ± 0.0104 m for the turning trajectory (mean ± sample standard deviation across five trials), while reducing localization drift and preventing the AMCL divergence observed under the tested mutual occlusion conditions.

## 1. Introduction

Autonomous mobile robots (AMRs) operating in a known map environment commonly combine two-dimensional LiDAR, odometry, and adaptive Monte Carlo localization (AMCL). AMCL represents pose uncertainty using a sequential Monte Carlo method [1], while LiDAR provides real-time environmental measurements [2]. When multiple AMRs travel in close formation, neighboring robots may simultaneously occlude observable static map features and produce time-varying robot body returns in the scans. Consequently, fewer valid environmental features remain available for scan-to-map matching, which may cause AMCL localization drift or divergence.

This study addresses this specific mutual occlusion degradation mechanism by proposing a Virtual-AMR LiDAR fusion method. The method identifies neighboring AMR contours, estimates the inter-robot SE(2) relationship, removes robot body returns, transforms the synchronized retained environmental measurements into a common Virtual-AMR coordinate frame, and supplies the fused virtual scan to an existing AMCL node. The focus is therefore on measurement construction before AMCL rather than on modifying the AMCL estimator itself. The objective also differs from collaborative SLAM methods that jointly estimate maps or pose graphs and from cooperative localization methods that exchange relative measurements or particle beliefs [3,4,5,6].

The proposed processing pipeline contains two complementary branches. The feature recognition and tracking branch uses median filtering, smoothing, an ROI, and a linear Kalman filter to estimate relative position. The relative angle branch uses KD-tree overlap matching and a dynamic angular search to estimate relative heading. The outputs of the two branches jointly establish the relative pose, which is then used to mask returns from the other AMR and fuse the available environmental LiDAR points. The resulting Virtual-AMR scan is supplied to AMCL.

The method was validated with two physical AMRs under two occlusion scenarios: a 4 m straight-line round trip and a turning trajectory involving position exchange and rotation. Each experimental configuration was repeated five times. In addition to standard AMCL and the complete Virtual-AMR method, branch-wise ablation was used to assess the respective effects of the relative angle estimation branch and the feature recognition/tracking branch. All validation was performed with this dual-AMR configuration in a known indoor map under controlled occlusion conditions.

The main contributions of this study are as follows:

1. A complete SE(2) coordinate transformation and time synchronization model is established, explicitly defining the measurement transformations and fusion conditions among the LiDAR, AMR base, map, and Virtual-AMR coordinate frames.
2. An AMCL-compatible Virtual-AMR measurement-construction pipeline is proposed. It integrates robot contour recognition, robot return removal, relative pose estimation, and virtual scan fusion without modifying the existing AMCL estimator.
3. Physical validation is performed using straight-line and turning experiments with two AMRs. Five repeated trials and branch-wise ablation compare standard AMCL, the individual processing branches, and the complete method to quantify localization error, failure rate, and computational performance under the tested conditions.

## 2. Related Work

This section focuses on four categories of work directly related to the proposed method: ROS 2 multi-robot communication architectures, AMCL localization in known maps, LiDAR preprocessing and feature matching, and cooperative localization/collaborative SLAM. The discussion explains how prior studies support the sensing, tracking, and matching modules and positions Virtual-AMR measurement reconstruction within the mutual occlusion problem.

### 2.1. ROS 2 Multi-Robot Communication Architecture

ROS provides publish/subscribe and service-based communication among nodes [7]. ROS 2 further employs the Data Distribution Service (DDS) as its underlying communication architecture and uses quality-of-service (QoS) settings to support data transmission and reliability control in distributed robotic systems [8,9]. In this study, ROS 2 transports timestamped LiDAR, odometry, and control information from the two AMRs, enabling scan synchronization, coordinate transformation, and Virtual-AMR fusion within a common communication framework. ROS 2 also provides the implementation foundation for the overall system.

### 2.2. AMCL and AMR Localization Architecture

For AMR navigation in a known map, LiDAR and odometry can be combined with map-based localization results to support subsequent path planning and motion control [10,11]. ROS/navigation studies have established implementation frameworks spanning map-based localization, local planning, and multi-robot applications [12,13,14,15]. These studies clarify the engineering role of AMCL in known map navigation, but its localization quality still depends on whether the current LiDAR scan contains sufficient and consistent environmental features for matching with the static map.

AMCL/MCL represents pose uncertainty using a particle filter and can use KLD sampling to adjust the particle count, balancing estimation quality and computational efficiency [16,17]. In the proposed architecture, AMCL remains a fixed map localization back end, while the study focuses on reconstructing its input observations. KD-trees, by contrast, are suitable for nearest-neighbor queries and searches in two-dimensional point sets [18]. The proposed method therefore uses a KD-tree to match the relative angle between scans from the two AMRs. This KD-tree matching estimates only relative heading and is not part of the core AMCL algorithm.

### 2.3. LiDAR Feature Processing and Point Set Matching

Studies related to the contour-processing branch of the proposed method include LiDAR target detection and tracking, multi-LiDAR obstacle/motion estimation, scan processing, and noise removal [19,20,21,22,23,24]. Corner points and geometric contours can serve as features for object recognition with two-dimensional LiDAR [25], while temporal information can stabilize target position estimates in tracking methods [26]. These studies support the use of filtering, contour features, and tracking to obtain the relative position of a neighboring AMR; however, these modules alone do not constitute Virtual-AMR fusion.

Work related to the relative angle branch includes KD-tree-accelerated point set matching, robust registration, and approximate nearest-neighbor search [27,28,29]. These methods provide efficient correspondence searches and geometric-matching tools. Building on them, the proposed method integrates relative position/angle estimation, AMR return removal, coordinate transformation, and reconstruction of a virtual scan from retained environmental points into a measurement pipeline before AMCL, thereby addressing the loss of scan-to-map information caused by mutual occlusion between two AMRs.

### 2.4. Cooperative Localization and Virtual-AMR Fusion

Existing cooperative localization methods typically exchange inter-robot range/bearing measurements, relative poses, or probabilistic beliefs, whereas collaborative SLAM methods exchange descriptors, keyframes, submaps, or pose graph constraints to improve team pose observability, global localization recovery, or map consistency [3,4,5,6]. In contrast, this study focuses on degradation at the measurement layer before AMCL: a neighboring AMR both occludes static map features and introduces dynamic robot body returns into the two-dimensional LiDAR scan. The proposed method therefore neither exchanges particle beliefs nor performs inter-robot loop closure or distributed pose graph optimization. Instead, it estimates the relative SE(2) relationship between two AMRs, removes AMR-related returns, transforms the usable environmental points from both AMRs into a common Virtual-AMR coordinate frame, and forms an AMCL-compatible virtual scan. Accordingly, this study defines the research gap as measurement reconstruction under mutual occlusion. Table 1 summarizes the comparison with related methods.

## 3. Multi-AMR Localization Under Mutual Occlusion

This section describes the multi-AMR AMCL localization architecture used in this study and the LiDAR measurement degradation caused by mutual occlusion between neighboring AMRs. Because the proposed Virtual-AMR method operates at the measurement construction layer before AMCL, this section first defines the standard AMCL baseline architecture and the mutual occlusion problem. Section 4 then presents the proposed LiDAR processing and virtual scan construction method.

### 3.1. Multi-AMR Localization System Architecture

This study uses AMCL on a known two-dimensional occupancy map as the baseline localizer for each physical AMR. Each AMR performs particle-based pose estimation using two-dimensional LiDAR and wheel odometry. Odometry provides motion prediction, while the LiDAR measurement model updates the particle weights [1,16,17]. In the standard baseline configuration, the two AMRs run AMCL independently. The proposed Virtual-AMR pipeline does not modify AMCL itself; instead, it reconstructs the LiDAR observation supplied to AMCL.

**Figure 1:**
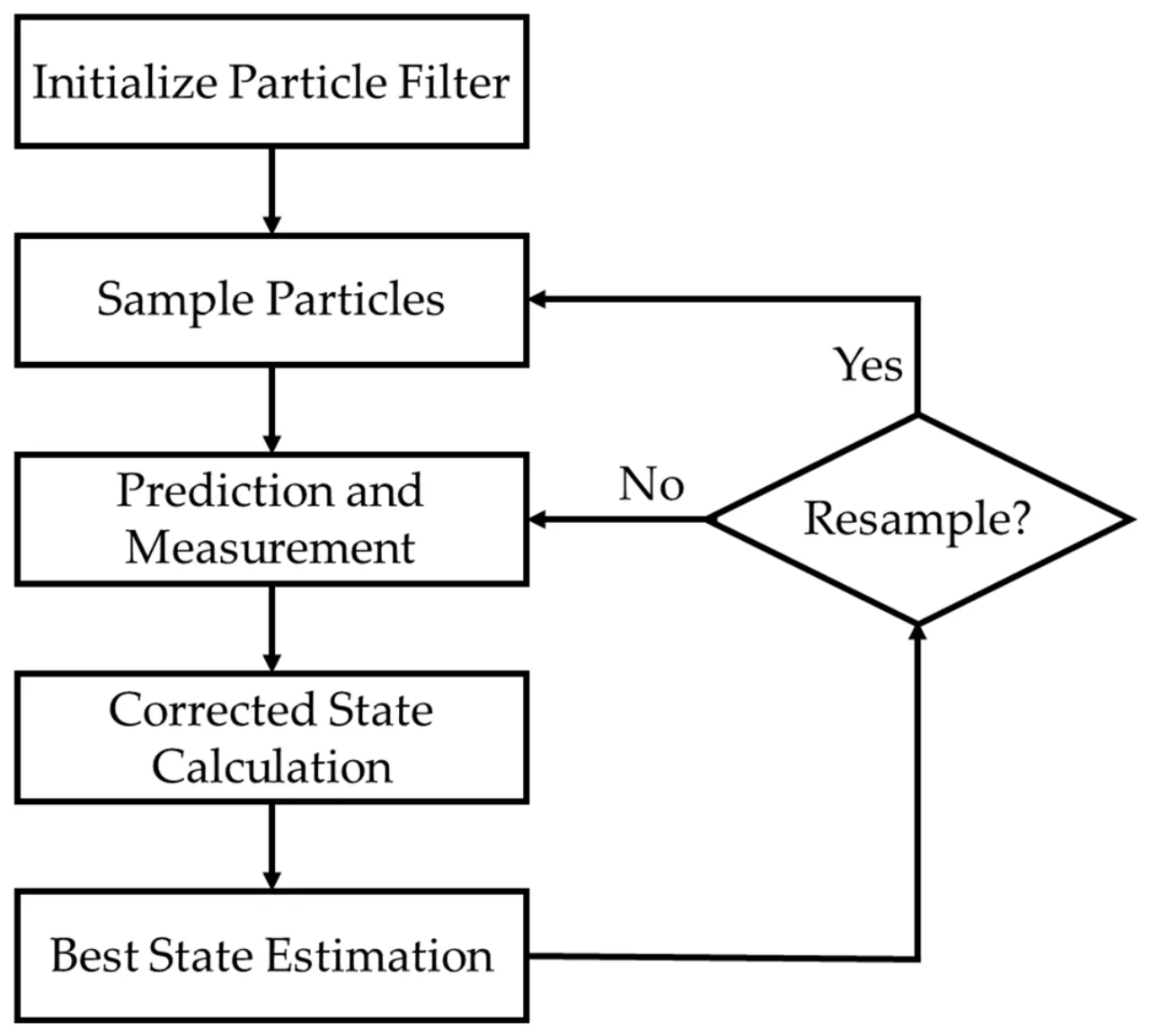
Prediction, measurement update, resampling decision, and state estimation steps of the particle filter localization process [30].

AMCL approximates the posterior distribution of the robot pose with a particle set. Its basic steps include motion prediction, LiDAR measurement updating, and resampling when required, as shown in Figure 1 [30]. Therefore, reduced consistency between the input scan and the static map directly affects the particle weight distribution and localization stability. This relationship forms the basis for analyzing the mutual occlusion problem in this study.

In the implemented system, AMCL performs map-based localization using an odometry motion model and a LiDAR likelihood field measurement model. This study does not seek to modify the AMCL algorithm itself; instead, it investigates how to improve the measurements supplied to AMCL when a neighboring AMR introduces dynamic returns and occludes static features. Existing indoor localization approaches may improve environmental distinctiveness by adding artificial landmarks [31]. In contrast, the proposed method requires no additional external landmarks and reconstructs a virtual environmental scan using the existing LiDAR information from the two AMRs.

The KD-tree is not part of the AMCL control core. In the proposed method, it serves only as the nearest-neighbor search structure for the relative angle estimation branch in Section 4.3 and is used to compare the overlap of LiDAR point sets from the two AMRs after coordinate transformation [18].

### 3.2. Mutual Occlusion Localization Problem

The AMCL LiDAR measurement model assumes that the principal environmental structures in the current scan correspond consistently to the existing static map. In a close dual-AMR formation, the other AMR has two adverse effects: it occludes walls or fixed objects that would otherwise be available for map matching, and it adds moving robot body returns that are absent from the static map. When the proportion of valid static features decreases, scan to map measurement consistency deteriorates. This may spread the particle weight distribution, cause pose drift, and, in severe cases, lead to the localization divergence observed in the experiments.

**Figure 2:**
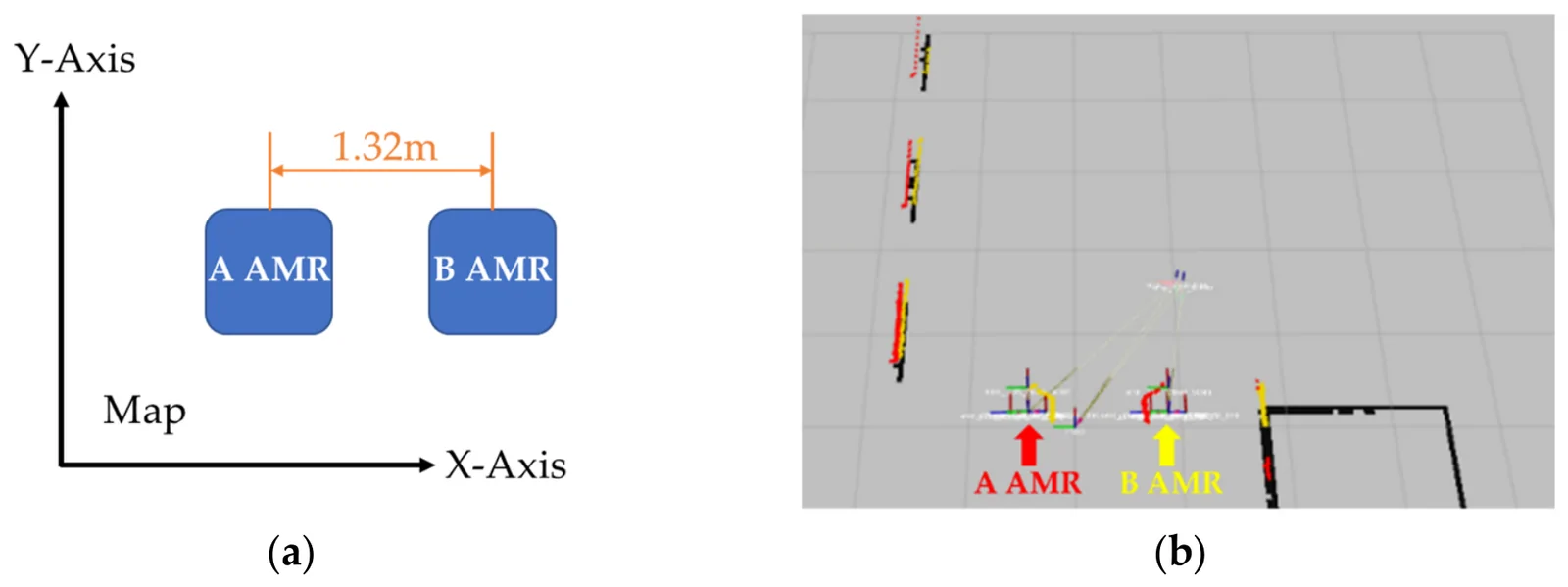
Relative positions of the two AMRs and their LiDAR measurements. (**a**) Relative positions of AMRs A and B; (**b**) mutual occlusion between the AMRs.

Figure 2 illustrates this problem through the relative positions of two AMRs. The red LiDAR points represent returns from the body of AMR B observed by AMR A, whereas the yellow LiDAR points represent returns from the body of AMR A observed by AMR B [2]. These body returns are not static map features and also occlude usable environmental measurements behind the robots. If the opposite side of an AMR lacks sufficient fixed geometric features, the information available to its individual AMCL process for map matching is further reduced. This study therefore defines the problem specifically as LiDAR measurement degradation caused by mutual occlusion rather than as general AMCL failure or localization in the presence of every type of dynamic obstacle.

Based on this problem definition, the Virtual-AMR layer is placed at the measurement input of standard AMCL to reconstruct an observation suitable for map matching under mutual occlusion. The following assumptions and validation boundaries define the conditions under which the method is applied:

1. The system uses a known two-dimensional occupancy map. Each two-dimensional LiDAR is mounted on the AMR base with fixed and calibrated planar extrinsic parameters, and the two AMRs move on a common horizontal plane.
2. A centralized fusion server receives timestamped LiDAR scans and pose data from the two AMRs. A valid fusion packet is formed only when the measurements satisfy the scan synchronization and data age conditions formally defined in Section 4.4.
3. At least one AMR must retain sufficient identifiable static environmental features at each fusion instant so that Virtual-AMR fusion can establish a reliable observation for map matching.
4. The physical validation in this study uses two AMRs and evaluates mutual occlusion, position exchange, and rotation through straight-line and turning trajectories.

## 4. Virtual-AMR Localization and Formation Control

This section formalizes the Virtual-AMR method and dual-AMR formation control. Section 4.1, Section 4.2 and Section 4.3 describe LiDAR preprocessing, contour feature recognition and tracking, and relative heading estimation, respectively. Section 4.4 defines the Virtual-AMR coordinates and virtual scan fusion, while Section 4.5 establishes the rigid formation motion model for the two AMRs. The emphasis is on the mathematical definitions, variables, and implementation relationships of the processing modules.

**Figure 3:**
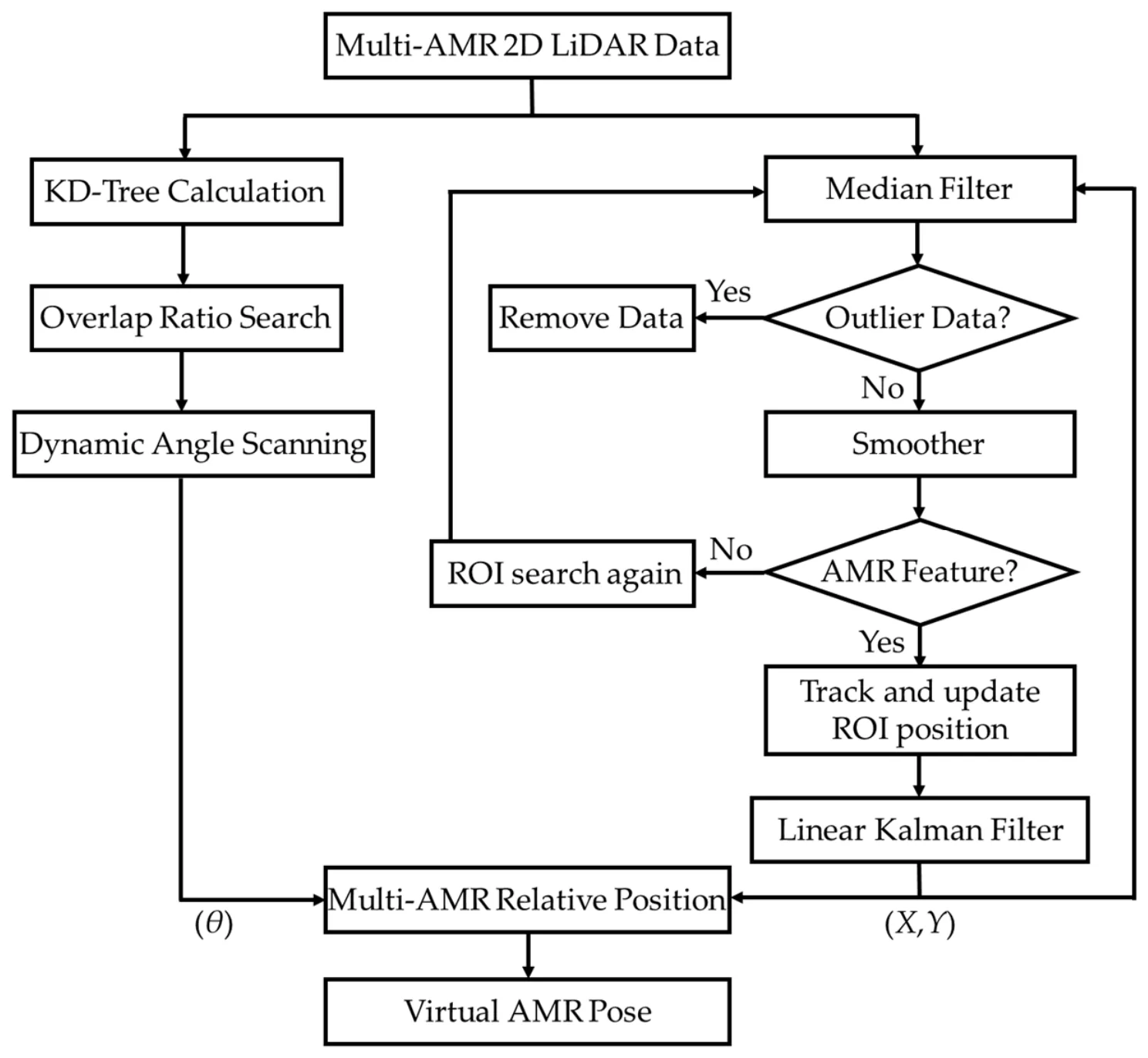
Localization algorithm pipeline.

The overall pipeline is shown in Figure 3. Timestamped LiDAR scans are first preprocessed and then passed through two complementary branches. The feature recognition and tracking branch uses contour geometry, an ROI, and a linear Kalman filter to estimate the relative position (*x*, *y*) between the AMRs. The relative angle branch uses KD-tree overlap matching to estimate the relative heading θ. Their outputs jointly form the inter-AMR relative pose (*x*, *y*, *θ*). The pipeline then performs time synchronization checks, coordinate transformation, AMR body return masking, and Virtual-AMR scan fusion. The fused virtual LiDAR measurement is supplied to the existing AMCL process for localization. The rigid formation motion model for the two AMRs is presented separately in Section 4.5.

### 4.1. LiDAR Data Acquisition and Preprocessing

Each two-dimensional LiDAR scan is represented by a scan index *ID*. The experiments use *ID_total_* = 655 measurement points per revolution. Equation (1) maps the scan index *ID* to the measurement angle *θ*(*ID*), and Equation (2) converts the measured range *r*(*ID*) into a planar point **p***^C^*(*ID*) in LiDAR coordinate frame *C*. Here, *θ_min_* and *θ_max_* are the starting and ending scan angles, *ID_total_* is the number of measurements used per revolution, and *x*(*ID*) and *y*(*ID*) are the coordinates of the LiDAR measurement point rather than the AMR localization coordinates in the map. The AMR center position is obtained through the contour recognition and geometric calculations described in Section 4.2.

(1) $$\theta (\mathrm{ID})=\theta_{\min}+\frac{\theta_{\max}-\theta_{\min}}{{\mathrm{ID}}_{\mathrm{total}}}*\mathrm{ID},$$

(2) $$p^{C}\left(\mathrm{ID}\right)=\left[\begin{array}{c} x(\mathrm{ID})\\ y(\mathrm{ID}) \end{array}\right]=\left[\begin{array}{cc} r(\mathrm{ID}) & 0\\ 0 & r(\mathrm{ID}) \end{array}\right]\left[\begin{array}{c} \cos(\theta \left(\mathrm{ID})\right)\\ \sin(\theta \left(\mathrm{ID})\right) \end{array}\right],$$

To improve the stability of contour segment extraction, the raw scan is first subjected to local outlier suppression and smoothing [24]. Median filtering reduces the effect of isolated measurements on the contour, while subsequent smoothing increases the consistency of local line segments. The processed point set serves only as input to AMR candidate contour extraction and KD-tree matching and does not alter the AMCL measurement model itself.

The proposed method uses a fixed 11-point median filtering window, as follows:

1. Data collection: Form *D* = {*d*_1_, *d*_2_, …, *d*_11_} from 11 consecutive range measurements.
2. Filtering: Calculate *d_median_* = median(*D*), where median(·) denotes the median of the 11-point window.
3. Data update: Replace the measurement at the center of the current window with d_median to obtain the filtered scan sequence.
4. Window shift: Move the window forward by one scan index, for example, *D*′ = {*d*_2_, *d*_3_, *d*_4_, …, *d*_12_}, and repeat Steps 2 and 3 until the current scan has been processed.

After median filtering, the original implementation sets *range* = 10 near *ID_start_* and uses the inclusive index interval from *ID_start_* to *ID_start_* + *range*, which therefore contains *range* + 1 = 11 coordinate points. Equation (3) calculates the smoothed coordinates over this interval in vector form, where **p***_i_* = [*x_i_*, *y_i_*]*^T^* is the ith planar LiDAR measurement point and **p***_s_*(*ID*) = [*x_s_*(*ID*), *y_s_*(*ID*)]*^T^* is the smoothed coordinate used for subsequent contour segment extraction:

(3) $$p_{s}\left(\mathrm{ID}\right)=\frac{1}{\mathrm{range}+1}\sum_{i=ID_{\mathrm{start}}}^{ID_{\mathrm{start}}+\mathrm{range}}p_{i},\;\;p_{i}={\left[x_{i},y_{i}\right]}^{T},$$

The smoothed point set is used to form candidate contour segments. The center position and tracking state of the target AMR are then estimated using the geometric feature recognition and Kalman tracking described in Section 4.2.

**Figure 4:**
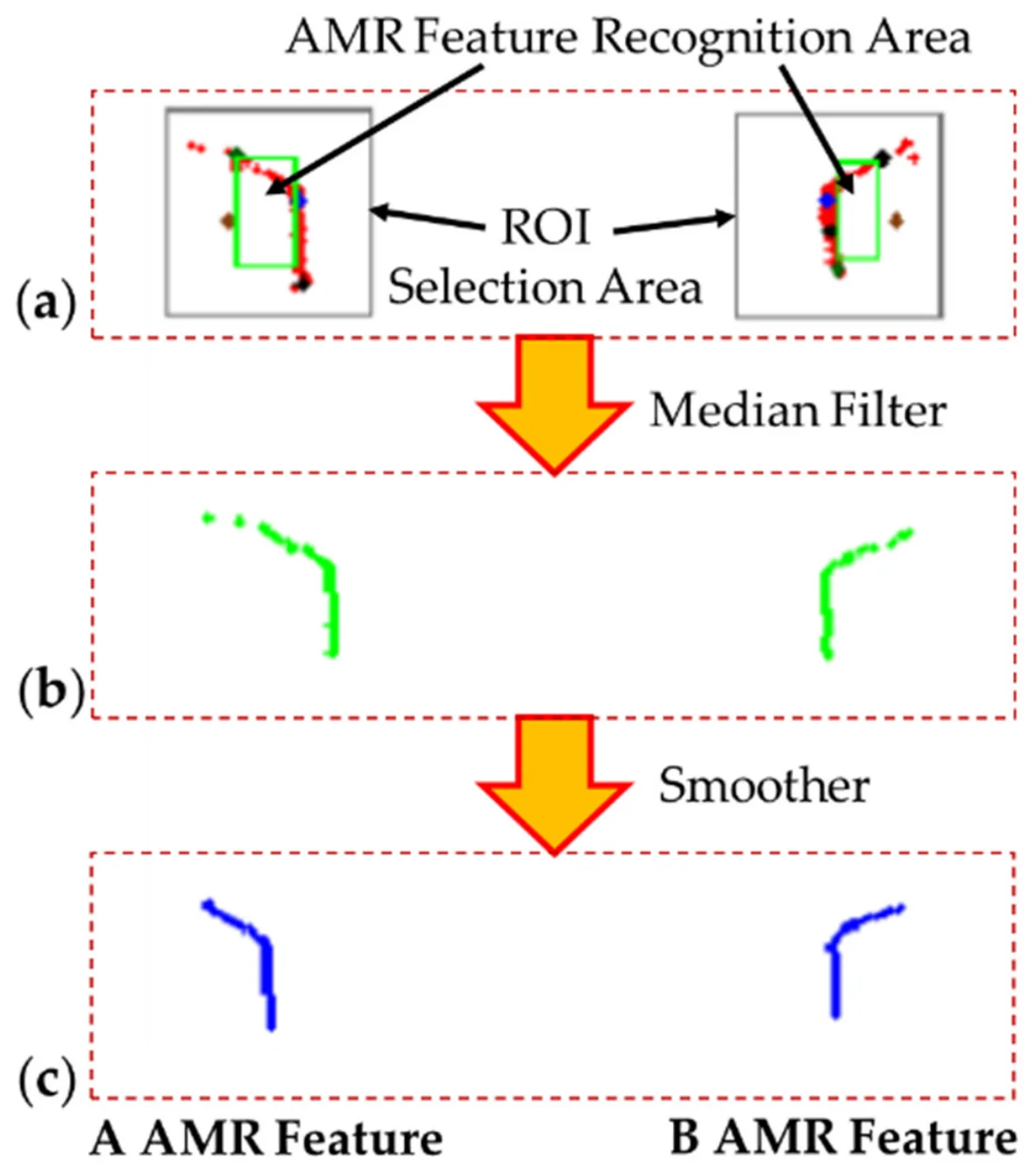
AMR contour-processing sequence: (**a**) raw ROI-selected contour points; (**b**) median-filtered points; and (**c**) smoothed line segments used for feature extraction.

For the scenario shown in Figure 2a, the filtering sequence is illustrated in Figure 4. Figure 4a shows the raw contour points selected by the ROI, Figure 4b shows the points after 11-sample median filtering, and Figure 4c shows the smoothed line segments used for subsequent feature extraction. The segment on the right is the contour of AMR B observed by AMR A, whereas the segment on the left is the contour of AMR A observed by AMR B.

### 4.2. AMR Contour Feature Recognition and Tracking

**Figure 5:**
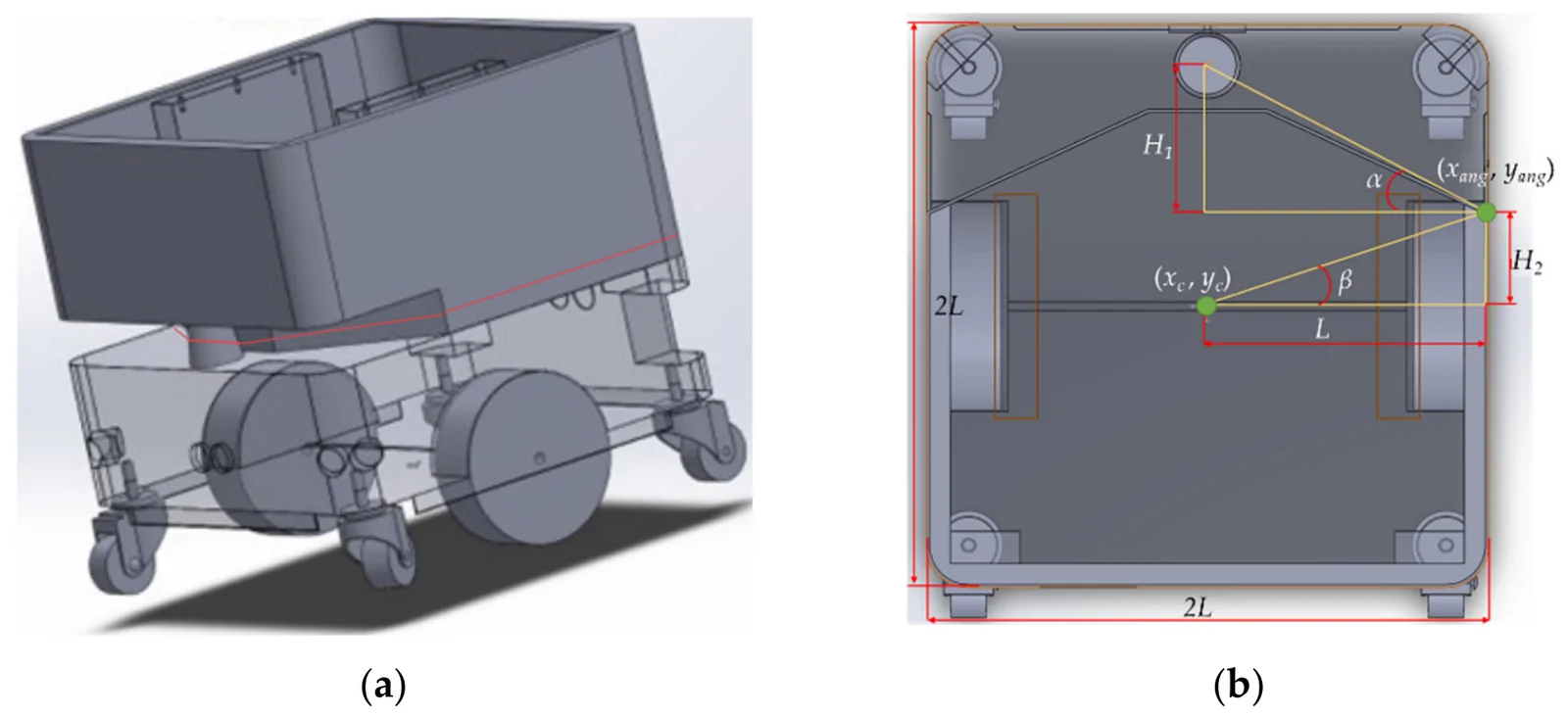
AMR platform geometry and LiDAR cross-section. (**a**) Three-dimensional chassis view; (**b**) top view defining *L*, *H*_1_, *H*_2_, *α*, *β*, the detected corner, and the center coordinate.

This section identifies the contour of a neighboring AMR and estimates its center position using the known chassis geometry. Figure 5 defines *L*, *H*_1_, and *H*_2_ for the subsequent contour geometry derivation. These dimensions and the LiDAR extrinsic parameters were obtained by direct geometric measurement of the physical platform.

The candidate contour obtained in Section 4.1 first initializes the ROI, after which *N* = 10 points on each side of a candidate corner are fitted by least squares. A candidate line segment is represented as *y_i_* = *ax_i_* + *b*, where a and b are the slope and intercept, respectively. Equation (4) calculates the slope a from N points (*x_i_*, *y_i_*). The proposed method fixes *N* = 10. The procedure is as follows:

1. Define the candidate region using the minimum and maximum scan indices in the ROI, with *ID_start_* denoting the search starting point.
2. Fit *y_i_* = *ax_i_* + *b* to the *N* scan points before and after the candidate corner.
3. Calculate the least squares slope a of each candidate line segment using Equation (4); *N* is set to 10:
  (4) $$\;a=\frac{N\left(\sum_{i=1}^{N}x_{i}y_{i}\right)-\left(\sum_{i=1}^{N}x_{i}\right)\left(\sum_{i=1}^{N}y_{i}\right)}{N\sum_{i=1}^{N}x_{i}^{2}-{\left(\sum_{i=1}^{N}x_{i}\right)}^{2}}.$$
4. Calculate the chassis feature angle *α* using Equation (5) and use it together with the candidate segment slopes to identify a corner consistent with the known AMR contour geometry:
  (5) $$\alpha =\mathrm{atan}2\left(H_{1},L\right),\;\;0<\alpha <\frac{\pi }{2}.$$

As shown in Figure 5b, comparison of the candidate segment slopes with the known chassis geometry yields the corner coordinate (*x_ang_*, *y_ang_*). Equations (5) and (6) define the characteristic angles *α* and *β* from the known dimensions *L*, *H*_1_, and *H*_2_. The angle *α* is used to evaluate geometric consistency of a candidate contour, while *β* corresponds to the magnitude of the angular offset used to infer the center from the known chassis geometry. In Equation (7), *θ_l_* is the direction of the fitted contour line; Δ*ϕ_geom_* is the signed geometric offset determined by whether the left or right symmetric corner is selected, with magnitude *β* from Equation (6); *ϕ_c_* is the direction from the AMR center to the detected corner; and *d_c_* is the fixed distance from the corner to the chassis center. The center coordinate (*x_c_*, *y_c_*) is then inferred from (*x_ang_*, *y_ang_*). Thus, *ϕ_c_* is obtained entirely from the observed contour direction and the known chassis geometry, without requiring prior knowledge of (*x_c_*, *y_c_*).

(6) $$\beta =\mathrm{atan}2\left(H_{2},\;L\right),\;\;0<\beta <\frac{\pi }{2}.$$

(7) $$\theta_{l}=\mathrm{atan}2\left(a,\;1\right)\;\;\phi_{c}=\theta_{l\;}+\Delta \phi_{\mathrm{geom}},\;\;d_{c}=\sqrt{L^{2}+H_{2}^{2}}\;\left[\begin{array}{c} x_{c}\\ y_{c} \end{array}\right]=\left[\begin{array}{c} x_{\mathrm{ang}}\\ y_{\mathrm{ang}} \end{array}\right]-d_{c}\left[\begin{array}{c} \cos\phi_{c}\\ \sin\phi_{c} \end{array}\right].$$

The contour tracker uses a linear constant-velocity Kalman filter. Let *i* denote the tracked AMR and *k* the discrete update index. Equation (8) defines and initializes the state ${\hat{x}}_{i,k}$, where *z_x,i,k_* and *z_y,i,k_* are the position measurements obtained from contour geometry. Because Kalman prediction and correction follow the standard linear estimation procedure, Equations (9a)–(9c), (9d) and (9e), and (9f)–(9h) describe state/covariance prediction, the position measurement model, and the Kalman update, respectively. Here, **F**(Δ*t*) is the state transition matrix; **P** and **Q** are the state and process noise covariance matrices; **H** is the measurement matrix; **v***_i,k_* is the measurement noise with covariance **R**; **K*_i_****_,k_* is the Kalman gain; **I** is the identity matrix; and the superscript “−” denotes the prior before the measurement update. Equation (10) dynamically adjusts the ROI half-widths *h_x,k_* and *h_y,k_* using velocity and the x/y position variances *P_xx,k_* and *P_yy,k_*_,_ where *γ* is the covariance expansion factor. The common AMR index *i* is omitted from Equation (10) for simplicity. The sampling interval, covariance matrices, and ROI settings are summarized below. If no valid contour measurement is available for the current packet, only the predicted state is retained. Three consecutive invalid synchronized packets trigger the research/recovery procedure.

(8) $${\hat{x}}_{i,k}={\left[\begin{array}{cccc} x_{i,k} & y_{i,k} & v_{x,i,k} & v_{y,i,k} \end{array}\right]}^{T}\;{\hat{x}}_{i,0}={\left[\begin{array}{cccc} z_{x,i,0} & z_{y,i,0} & 0 & 0 \end{array}\right]}^{T},\;\;P_{i,0}=P_{0}.$$

(9a) $${{\hat{x}}_{i,}^{\text{-}}}_{k}=F\left(\Delta t\right){\hat{x}}_{i,k-1}$$

(9b) $$P_{i,k}^{-}=F\left(\Delta t\right)P_{i,k-1}F^{T}\left(\Delta t\right)+Q$$

(9c) $$F\left(\Delta t\right)=\left[\begin{array}{cccc} 1 & 0 & \Delta t & 0\\ 0 & 1 & 0 & \Delta t\\ 0 & 0 & 1 & 0\\ 0 & 0 & 0 & 1 \end{array}\right]$$

(9d) $$z_{i,k}=H{\hat{x}}_{i,k}^{-}+v_{i,k},\;v_{i,k}∼N\left(0,R\right)$$

(9e) $$H=\left[\begin{array}{cccc} 1 & 0 & 0 & 0\\ 0 & 1 & 0 & 0 \end{array}\right]$$

(9f) $$K_{i,k}=P_{i,k}^{-}H^{T}{\left(HP_{i,k}^{-}H^{T}+R\right)}^{-1}$$

(9g) $${\hat{x}}_{i,k}={\hat{x}}_{i,k}^{-}+K_{i,k}\left(z_{i,k}-H{\hat{x}}_{i,k}^{-}\right)$$

(9h) $$P_{i,k}=\left(I-K_{i,k}H\right)P_{i,k}^{-}$$

(10) $$h_{x,k}={\;h}_{x,0}+\left|v_{x,k}\right|\Delta t+\gamma \sqrt{P_{\mathrm{xx},k}}\;h_{y,k}={\;h}_{y,0}+\left|v_{y,k}\right|\Delta t+\gamma \sqrt{P_{\mathrm{yy},k}}.$$

### 4.3. Relative Angle Estimation

**Figure 6:**
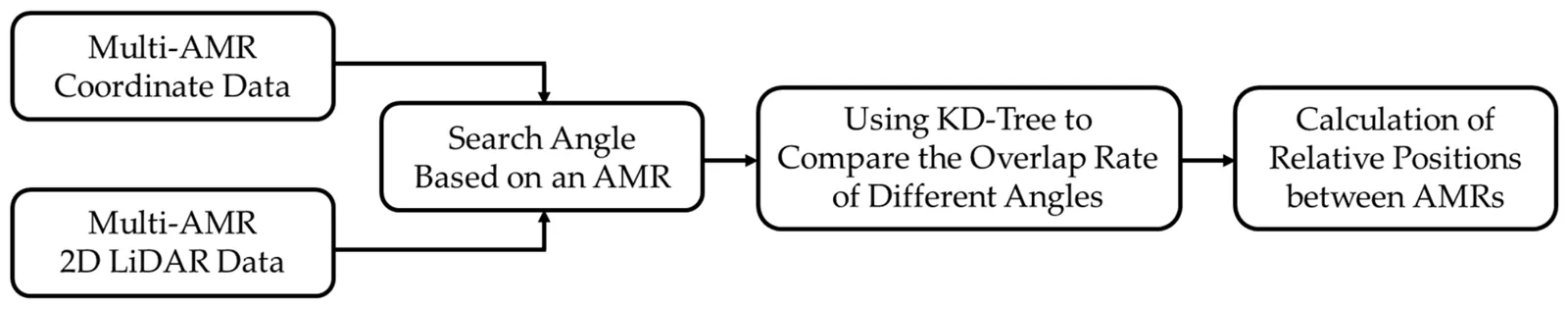
KD-tree method for relative angle estimation.

The relative angle branch compares the filtered LiDAR point sets from the two AMRs after coordinate transformation using a KD-tree nearest-neighbor search [27,28], as shown in Figure 6. Let *i*, *j* ∈ {A, B}, *i* ≠ *j*, denote the source and target AMRs, and let *S_i_* and *S_j_* denote their preprocessed LiDAR point sets, respectively. Each scan contains 655 points. For each candidate source point, the target search is restricted to a window of ±10 points around the corresponding scan index, and a post-filtering distance threshold of 3 mm is used to determine geometric correspondence. Before candidate rotation searching, the two point sets are compensated for translation using the available relative position estimate ${\hat{t}}_{\mathrm{ij}}$ = [${\hat{x}}_{\mathrm{ij}}$, ${\hat{y}}_{\mathrm{ij}}$]^T^. This estimate may be obtained from the feature recognition and tracking branch or from the available localization outputs, depending on the active processing configuration; the specific sources used in each experimental configuration are defined in Section 5.3.5. After translation compensation, an overlap score is calculated for each candidate rotation *δθ*, and the rotation that maximizes this score is selected as the relative heading estimate.

Equation (11) calculates the scan overlap score *s*(*δθ*) from *N_p_* candidate points, where **p***_k_* ∈ *S_i_* is the *k*th source point; **q** ∈ *S_j_* is a candidate corresponding point in the target set; **R**(*δθ*) is the two-dimensional rotation matrix; ${\hat{t}}_{\mathrm{ij}}$ is the translation compensation; *ε_d_* is the nearest-neighbor distance gate; and 1[·] is the indicator function. Equation (12) selects from the candidate angle set *Ω* the optimal angle *δθ_opt_* that maximizes *s*(*δθ*). This value is defined as the relative heading estimate for the source–target pair, ${\hat{\theta }}_{\mathrm{ij}}$ = *δθ_opt_*, and is consistently denoted ${\hat{\theta }}_{\mathrm{AB}}$ when establishing the Virtual-AMR coordinates. The value *ε_d_* = 3 mm is an empirical geometric consistency threshold for the filtered and transformed point sets, not the raw ranging accuracy of the TiM551 [32]. Similarly, 0.1° is the numerical increment of the relative angle optimization rather than the physical angular resolution of the sensor. The sensitivity of the 3 mm and 0.1° settings is analyzed in Section 5.2.3.

(11) $$s\left(\delta \theta \right)=\frac{1}{N_{p}}\sum_{k=1}^{N_{p}}1\;\left[\min_{q\in S_{j}}{∥R\left(\delta \theta \right)p_{k}+{\hat{t}}_{ij}-q∥}_{\;2}<\varepsilon_{d}\right].$$

(12) $$\delta \theta_{\mathrm{opt}}=\underset{\delta \theta \in \Omega }{\arg\;\max}\;s\left(\delta \theta \right).$$

**Figure 7:**
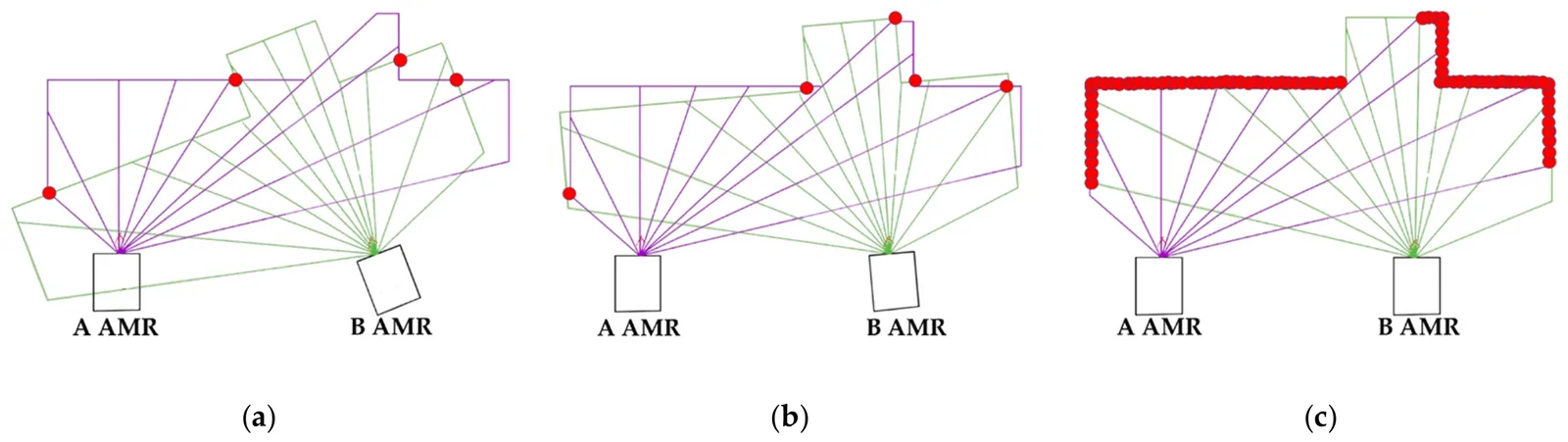
Coordinate matching of AMR B with respect to AMR A. (**a**) AMR B rotated by 20°; (**b**) AMR B rotated by 10°; and (**c**) AMR B rotated by 0°.

Figure 7 illustrates how the overlap score changes among three candidate rotations. When the relative angle between the two point sets approaches the correct value, more corresponding points satisfy *ε_d_*, and the overlap score in Equation (11) increases. The candidate angle in Figure 7c has the highest overlap among the three examples. The implemented estimator uses Equation (12) to select the maximum overlap score over the search set *Ω*.

The principal parameters of the preceding method and equations are summarized in Table 2.

The process covariance matrix **Q** was derived from the discrete constant-velocity model using the sampling interval Δ*t* = 0.1 s and an acceleration standard deviation of 0.05 m/s^2^, selected as an engineering setting based on the maximum commanded acceleration used in the experiments. The measurement covariance **R** = diag(4.0 × 10^−4^, 4.0 × 10^−4^) m^2^ corresponds to an assumed position measurement standard deviation of 0.02 m selected as an engineering setting for contour center measurement uncertainty. The initial covariance **P**_0_ represents standard deviations of 0.05 m for position and 0.10 m/s for velocity. The initial ROI half-widths of 0.25 m were selected from the 0.20 m chassis half-width with an additional 0.05 m detection margin, while *γ* = 2.0 expands the ROI according to approximately two standard deviations of the predicted position uncertainty. These parameters were kept fixed in all experiments.

### 4.4. Virtual-AMR Coordinate Construction and LiDAR Fusion

Let *M* be the map coordinate frame, *B_i_* the base frame of AMR *i*, *L_i_* its LiDAR frame, and *V* the Virtual-AMR frame. $T_{B}^{A}$ transforms a point expressed in frame B into frame A. In the experiments, *i* ∈ {*A*, *B*}. Equation (13) defines a general SE(2) homogeneous transformation, where *ψ* is the planar rotation and *t_x_* and *t_y_* are the translations. At each valid fusion timestamp *t_k_*, the relative position (${\hat{x}}_{\mathrm{AB}}$, ${\hat{y}}_{\mathrm{AB}}$) from Section 4.2 and the relative heading ${\hat{\theta }}_{\mathrm{AB}}$ from Section 4.3 are combined into the relative transform ${\hat{T}}_{B_{B}}^{B_{A}}$ in Equation (14), which transforms quantities from AMR B base frame *B_B_* to AMR A base frame *B_A_*. Equation (15) defines the scan synchronization and data age conditions. Equations (16)–(18) establish the instantaneous Virtual-AMR geometry and the transformations from both LiDAR frames to *V*. Equation (19) then constructs the fused environmental point set after AMR body returns have been removed.

In Equation (15), *t_i_* and *t*_rx*,i*_ are, respectively, the LiDAR scan timestamp of AMR *i* and its reception time at the fusion server; Δ*t_sync,max_* and *τ_age,max_* limit the inter-scan timestamp difference and data age. To simplify notation, some common time indices *k* are omitted from Equations (16)–(19); all unindexed ${\hat{x}}_{\mathrm{AB}}$, ${\hat{y}}_{\mathrm{AB}}$, and ${\hat{\theta }}_{\mathrm{AB}}$ values correspond to the same fusion instant *t_k_*. The quantities *w_A_* and *w_B_* are fusion weights; $x_{V}^{A}$, $y_{V}^{A}$, and $\psi_{V}^{A}$, express *V* in frame *B_A_*; and $T_{V}^{B_{A}}$ transforms *V* to *B_A_*. In Equation (18), $T_{L_{i}}^{B_{i}}$ is the fixed calibrated extrinsic transform from the LiDAR frame to the base frame. In Equation (19), *S_i_*(*t_i_*), *M_i_*, **p**, and *S_V_*(*t_k_*) are, respectively, the preprocessed point set in *L_i_*, the AMR body return mask in the same frame, a single LiDAR point, and the fused point set in *V*.

(13) $$T_{B}^{A}=\left[\begin{array}{ccc} \cos\psi & -\sin\psi & t_{x}\\ \sin\psi & \cos\psi & t_{y}\\ 0 & 0 & 1 \end{array}\right]\in \mathrm{SE}\left(2\right).$$

(14) $${\hat{T}}_{B_{B}}^{B_{A}}\left(t_{k}\right)=\mathrm{SE}\left(2\right)\;\left({\hat{x}}_{\mathrm{AB},k},\;{\hat{y}}_{\mathrm{AB},k},\;{\hat{\theta }}_{\mathrm{AB},k}\right).$$

(15) $$\underset{i}{\max}\left(t_{i}\right)-\underset{i}{\min}\left(t_{i}\right)\;\leq \;\Delta t_{\mathrm{sync},\max},\;\underset{i}{\max}\left(t_{\mathrm{rx},i}-t_{i}\right)\;\leq \;\tau_{\mathrm{age},\max}.$$

(16) $$x_{V}^{A}=\frac{w_{B}{\hat{x}}_{\mathrm{AB}}}{w_{A}+{\;w}_{B}},\;\;y_{V}^{A}=\frac{w_{B}{\hat{y}}_{\mathrm{AB}}}{w_{A\;}+{\;w}_{B}}\;\psi_{V}^{A}=\mathrm{atan}2\left(w_{B}\sin{\hat{\theta }}_{\mathrm{AB}},\;w_{A}+w_{B}\cos{\hat{\theta }}_{\mathrm{AB}}\right).$$

(17) $$T_{V}^{B_{A}}=\mathrm{SE}\left(2\right)\left(x_{V}^{A},\;y_{V}^{A},{\;\psi }_{V}^{A}\right).$$

(18) $$T_{L_{A}}^{V}={\left(T_{V}^{B_{A}}\right)}^{-1}T_{L_{A}}^{B_{A}}\;T_{L_{B}}^{V}={\left(T_{V}^{B_{A}}\right)}^{-1}{\hat{T}}_{B_{B}}^{B_{A}}T_{L_{B}}^{B_{B}}.$$

(19) $$S_{V}\left(t_{k}\right)=\underset{i\in \{A,B\}}{⋃}\left\{T_{L_{i}}^{V}p\;:\;p\;\in S_{i}\left(t_{i}\right),\;p\notin M_{i}\right\}.$$

Both the straight-line and turning experiments use *w_A_* = *w_B_* = 1. Therefore, *V* lies at the spatial midpoint of the current relative configuration of the two AMRs, while heading *ψ_V_* is obtained by circular averaging; no temporal averaging is performed. Equations (16)–(18) define the instantaneous geometry of the scan fusion frame. Separately, AMCL estimates and outputs the localization result ${\hat{T}}_{V}^{M}$ in map coordinates from the Virtual-AMR scan constructed in Equation (19). The Virtual AMR is therefore not a third physical robot, and the geometric midpoint in Equation (16) is not the final AMCL localization estimate reported in this study.

**Figure 8:**
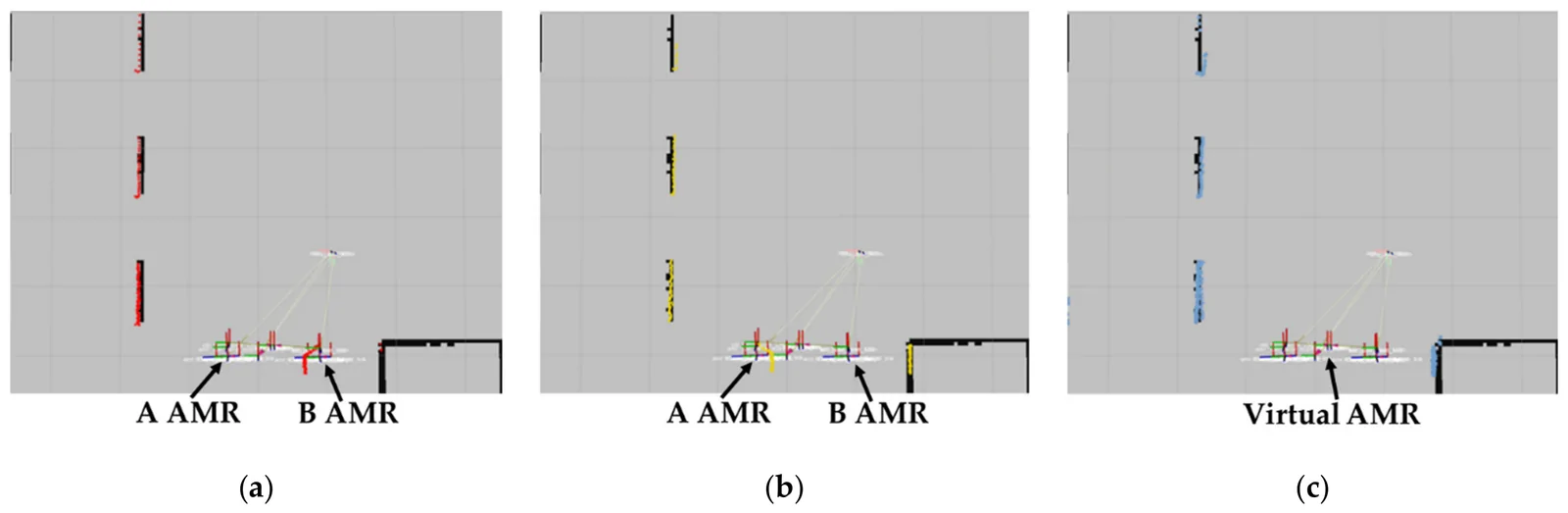
Generation of a Virtual-AMR LiDAR scan from the surrounding physical AMR LiDAR data. (**a**) LiDAR scan from AMR A; (**b**) LiDAR scan from AMR B; and (**c**) Virtual-AMR LiDAR scan.

1. First, Equation (15) is used to verify that the two LiDAR packets satisfy the time synchronization and data age conditions. The relative position from Section 4.2 and the relative heading from Section 4.3 are then combined into ${\hat{T}}_{B_{B}}^{B_{A}}$ in Equation (14).
2. Equations (16)–(18) establish the current local Virtual-AMR geometry and the transformations from both LiDAR frames to *V*. Simultaneously, the tracked neighboring AMR center, relative heading, and known chassis outline are used to construct the AMR body mask *M_i_*; returns within that chassis footprint are excluded from the environmental point set.
3. The valid environmental points in *S_i_*\*M_i_* are fused according to Equation (19). Each point (*x*, *y*) in *V* is converted back to polar coordinates using *θ* = atan2(*y*, *x*) and *r* = $\sqrt{(x^{2}+y^{2})}$ and assigned to an angular bin in a Virtual LaserScan message. If multiple valid points fall in the same bin, the minimum range is retained. Bins without valid points are treated as no-return bins according to the LaserScan convention. The resulting virtual scan shown in Figure 8 is then supplied to the existing AMCL node.

**Figure 9:**
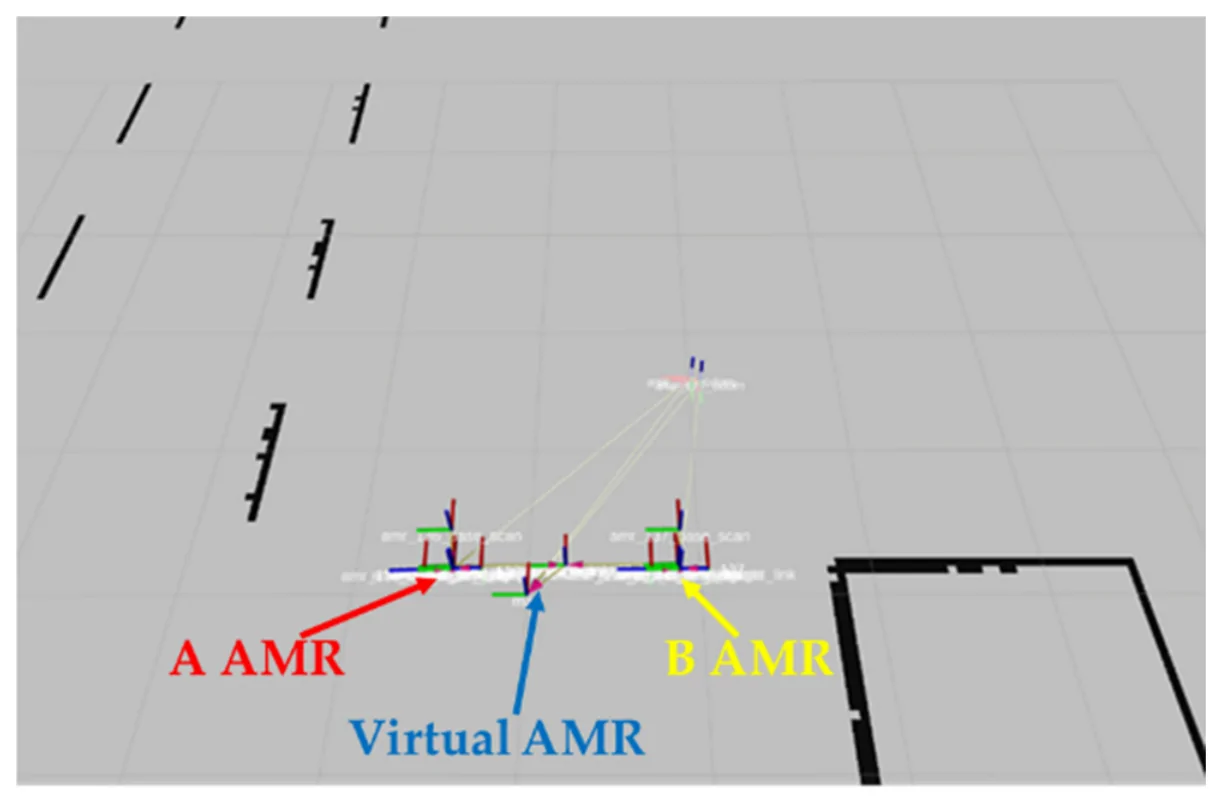
TF relationships of the dual-AMR configuration.

The output of the Virtual-AMR layer is the fused Virtual LaserScan and its corresponding TF, not an additional localization estimator. AMCL performs its existing prediction/measurement update using the Virtual-AMR scan and outputs ${\hat{T}}_{V}^{M}$. The relative TF relationships between the two physical AMRs and *V*, as shown in Figure 9, are obtained from the fusion geometry in Equations (14)–(18) and are used by the dual-AMR formation controller described in Section 4.5.

### 4.5. Dual-AMR Rigid Formation Control

The formation controller is restricted to two heading-aligned AMRs and uses the Virtual-AMR center as the rigid formation reference. Let *r_A_* = [0, +*R*]^T^ and *r_B_* = [0, −*R*]^T^ denote the lateral offsets of AMRs A and B relative to the Virtual AMR, where *R* is the lateral distance from each AMR to the Virtual-AMR center, i.e., half the center-to-center distance between the two AMRs. The quantities *v_x,V_* and *ω_V_* are the reference linear and angular velocities of the Virtual AMR, while *v_x,A_*, *v_x,B_*, *ω_A_*, and *ω_B_* are the corresponding velocities of the two physical AMRs. Applying the planar rigid body velocity relationship *v_i_* = *v_V_* + *ω_V_* × *r_i_* yields the dual-AMR linear and angular velocity relationships in Equations (20a)–(20d):

(20a) $$v_{x,A}=v_{x,V}-\omega_{V}R.$$

(20b) $$\omega_{A}=\omega_{V}.$$

(20c) $$v_{x,B}=v_{x,V}+\omega_{V}R.$$

(20d) $$\omega_{B}=\omega_{V}.$$

When *ω_V_* = 0, Equations (20a) and (20c) give *v_x,A_* = *v_x,B_* = *v_x,V_*, while Equations (20b) and (20d) give *ω_A_* = *ω_B_* = 0, corresponding to the straight-line condition. When *ω_V_* ≠ 0, Equations (20a) and (20c) apply opposite ±*ω_V_R* linear velocity corrections to the inner and outer AMRs, while Equations (20b) and (20d) maintain *ω_A_* = *ω_B_* = *ω_V_*, corresponding to the turning condition.

## 5. Experimental Results

This section evaluates the proposed Virtual-AMR method under controlled mutual occlusion conditions involving two AMRs. For clarity, the four branch-wise test configurations are denoted as follows: T1 is the standard individual-scan AMCL/TF baseline; T2 activates only the relative angle estimation branch; T3 activates only the feature recognition/tracking branch; and T4 activates both branches to construct the complete Virtual-AMR scan. The complete settings and the sources of relative position and heading for each configuration are detailed in the branch-wise ablation in Section 5.3.5. The evaluation covers (1) virtual scan construction and relative angle estimation; (2) CPU utilization, end to end latency, and sensitivity to KD-tree parameters; (3) localization error, particle cloud dispersion, and internal consistency under the 4 m straight-line condition; and (4) branch-wise ablation under the turning condition. Each T1–T4 configuration was evaluated in five independent repeated trials. Section Evaluation Protocol and Metrics defines the evaluation reference, statistical metrics, and evidentiary boundaries.

### 5.1. Experimental Platform and Environment

**Figure 10:**
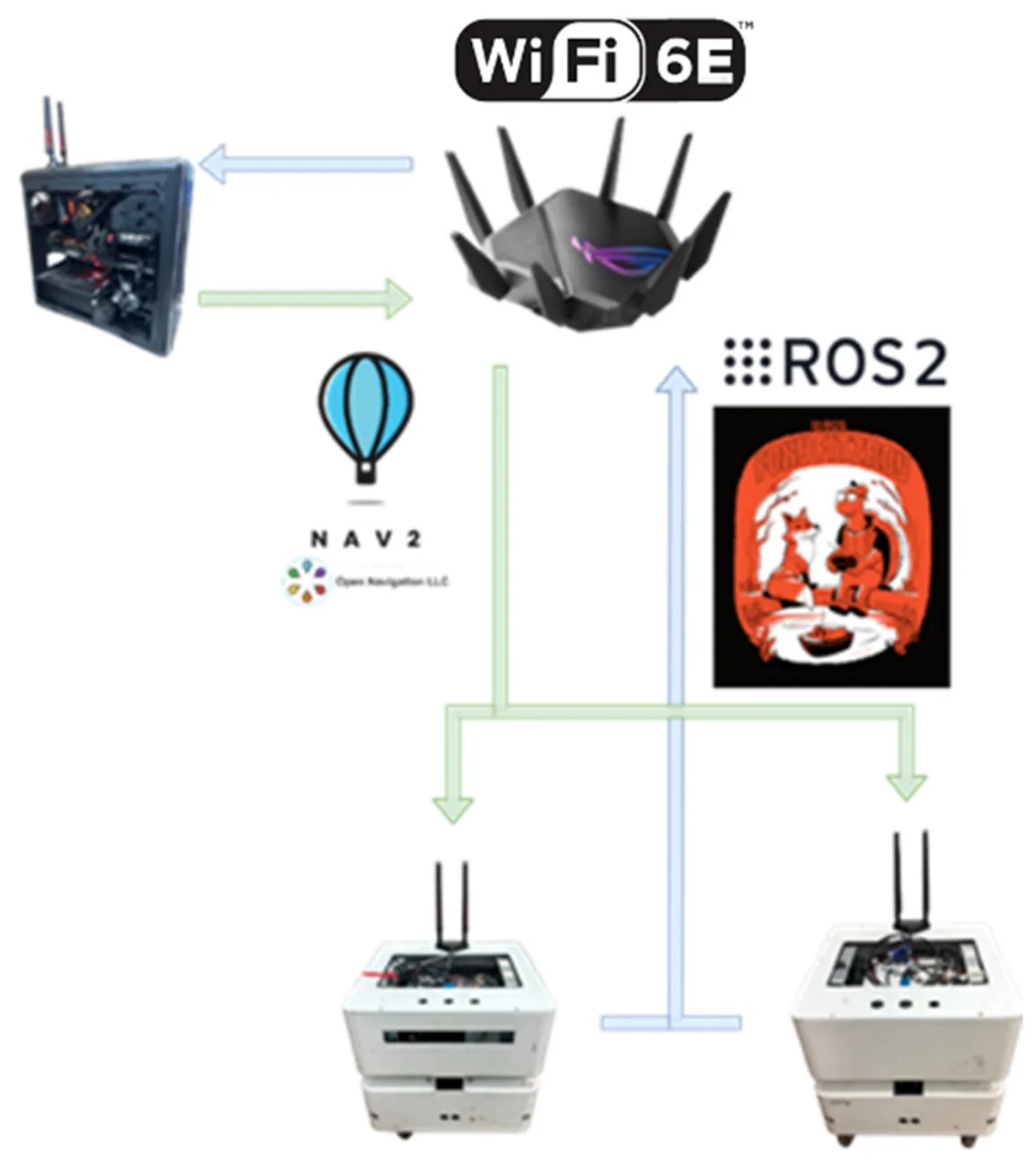
Experimental system architecture.

**Figure 11:**
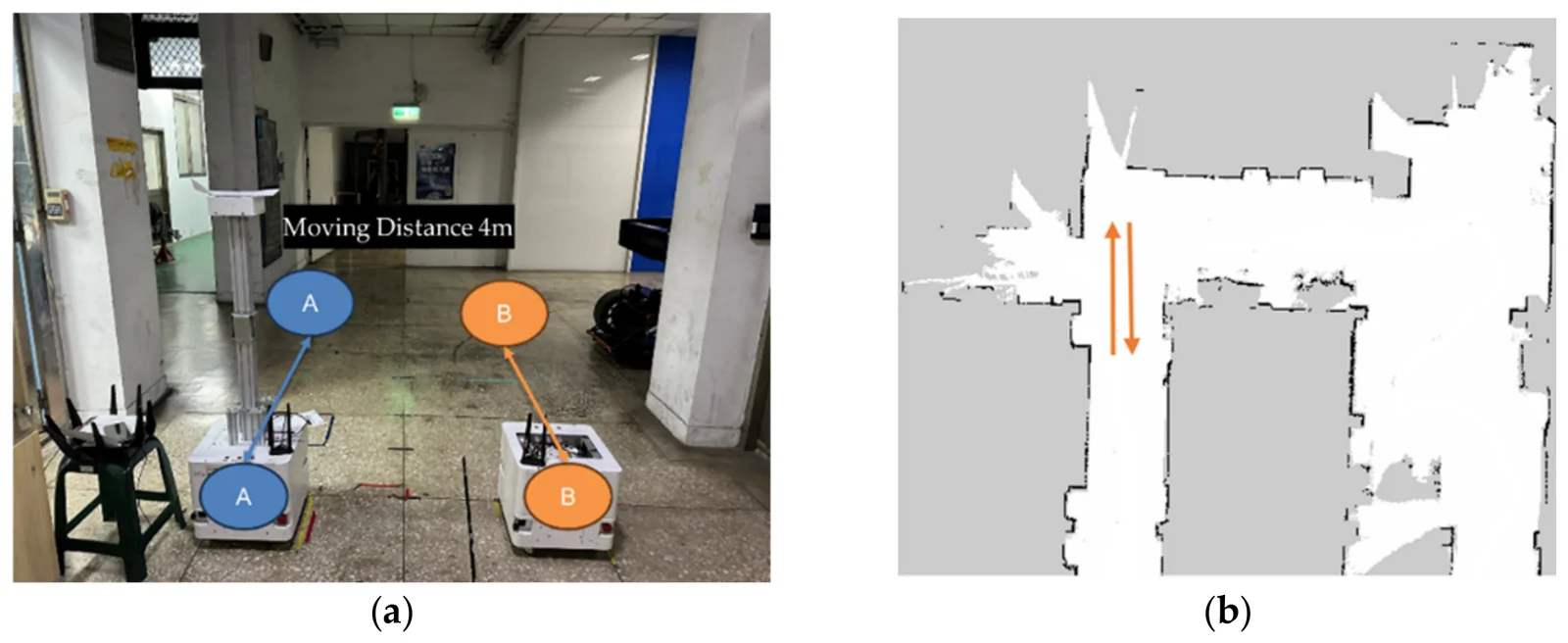
Experimental area and motion trajectory. (**a**) Experimental area; (**b**) motion trajectory.

The experimental system uses a Wi-Fi 6E communication architecture and two physical AMRs, as shown in Figure 10. The data link transmits LiDAR and odometry measurements to the server, which publishes linear velocity and z-axis angular velocity commands to AMRs A and B. The hardware specifications are listed in Table 3. The test site is a 3.1 m × 10 m indoor open area with floor tiles measuring approximately 0.84 m × 0.86 m. The initial positions and straight-line direction of the two AMRs are shown in Figure 11. No appreciable tire–floor slip was assumed during the experiments. Section Evaluation Protocol and Metrics describes how the reference trajectory was established and defines the corresponding evaluation boundaries.

The two AMRs use identical LiDAR mounting geometry. The LiDAR extrinsic parameters were obtained by measuring the chassis mounting geometry: the LiDAR is located on the longitudinal chassis centerline at $x_{L}^{B}$ = *H*_1_ + *H*_2_ = 0.075 + 0.100 = 0.175 m and $y_{L}^{B}$ = 0, and its zero-degree direction is aligned with the forward chassis axis. Therefore, **T***ᴮ_L_* = (0.175 m, 0, 0°).

**Figure 12:**
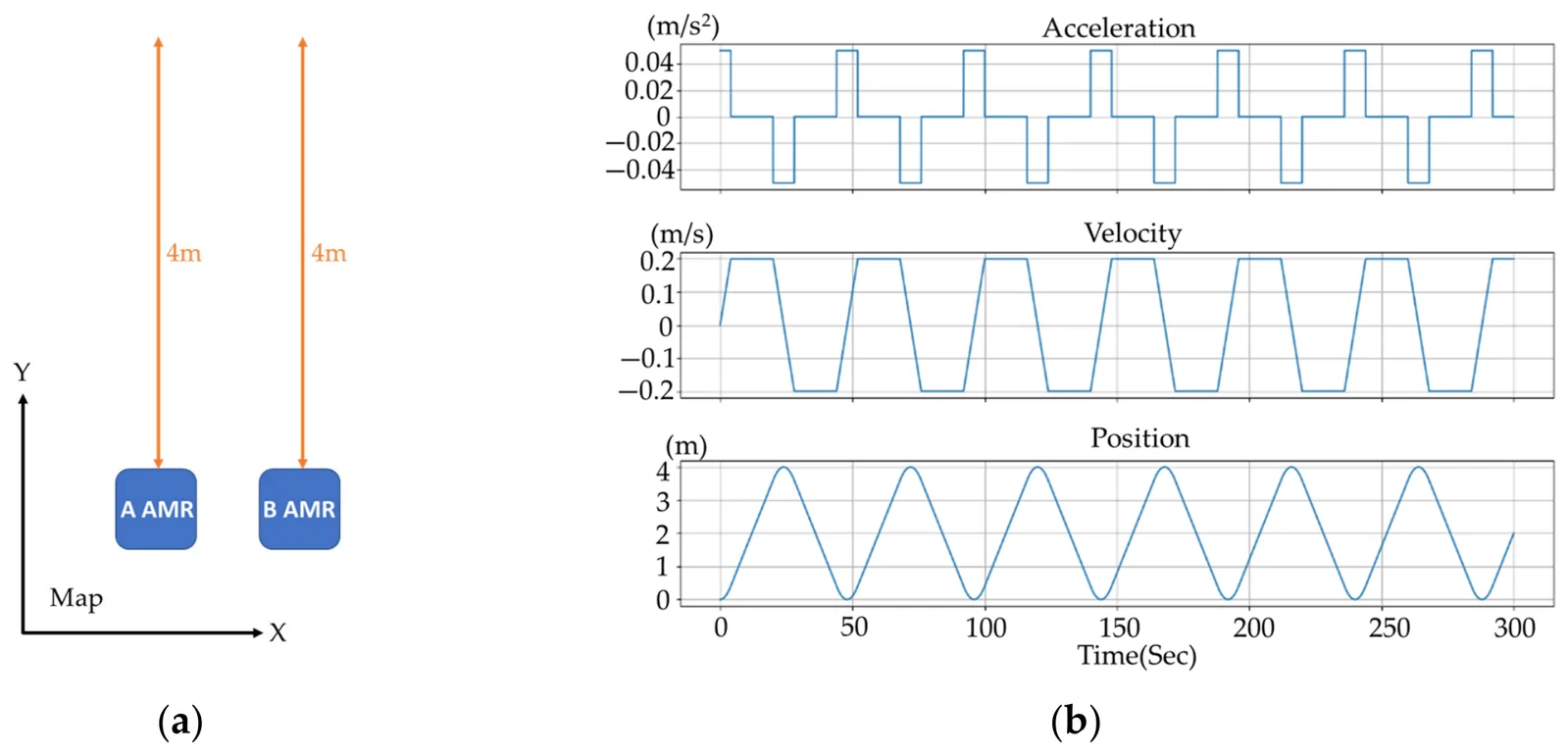
Experimental path planning. (**a**) Motion pattern; (**b**) position, velocity, and acceleration profiles.

In the straight-line experiments, the two AMRs traveled back and forth along a 4 m path, as shown in Figure 12. Their maximum linear velocity, maximum acceleration, and angular velocity were limited to 0.22 m/s, 0.05 m/s^2^, and 0.2 rad/s, respectively. All configurations used the same motion parameters and position control strategy to reduce the influence of control condition differences on the comparison. The experiments simultaneously recorded the AMCL pose, particle cloud, odometry, and algorithm execution information. Each T1–T4 configuration under the straight-line condition was evaluated in five independent repeated trials.

#### Evaluation Protocol and Metrics

Let ${\hat{p}}_{k}$ = [${\hat{x}}_{k}$, ${\hat{y}}_{k}$]^T^ denote the localization output and ${\hat{p}}_{\mathrm{ref},k}$ = [${\hat{x}}_{\mathrm{ref},k}$, ${\hat{y}}_{\mathrm{ref},k}$]^T^ the reference trajectory used in this study, obtained by aligning the planned path with displacement information from the motor encoders. Equation (21) defines the x-axis error *e_x,k_*, y-axis error *e_y,k_*, and two-dimensional position error *e*_2*D,k*_ for the *k*th valid sample. Equation (22) defines MAE and RMSE using the same equation set. The maximum absolute error E_max, median error E_med, and 95th-percentile error E_P95 are obtained directly from the same pooled error sample set as the maximum absolute value, median, and 95th percentile, respectively. Each of the T1–T4 configurations in the straight-line and turning experiments was evaluated in five independent repeated trials. For each configuration, reference alignment was first performed separately for all five trials, after which all valid samples were combined into a single pooled sample set. The metrics were then calculated using Equations (21) and (22) and the summary statistics defined above. Thus, *N* is the total number of valid samples across the five trials; it is not an average of five trial-level metrics. Because the reference trajectory is an internal comparison baseline, the results are described in terms of tracking error and localization stability and are not interpreted as absolute localization accuracy verified by an independent external measurement system. Particle cloud dispersion is used to observe the spread of the AMCL posterior, and odometry-related signals are used to examine internal motion consistency. Both are supplementary analyses rather than independent localization accuracy metrics.

(21) $$e_{x,k}={\hat{x}}_{k}-x_{\mathrm{ref},k},\;\;e_{y,k}={\hat{y}}_{k}-y_{\mathrm{ref},k}\;e_{2D,k}=\sqrt{e_{x,k}^{2}+e_{y,k}^{2}}.$$

(22) $$\mathrm{MAE}=\frac{1}{N}\sum_{k=1}^{N}\left|e_{k}\right|.\;\mathrm{RMSE}=\sqrt{\frac{1}{N}\sum_{k=1}^{N}e_{k}^{2}}.$$

### 5.2. Virtual Scan Construction and Relative Angle Estimation

#### 5.2.1. AMR Recognition and Virtual Scan Construction

**Figure 13:**
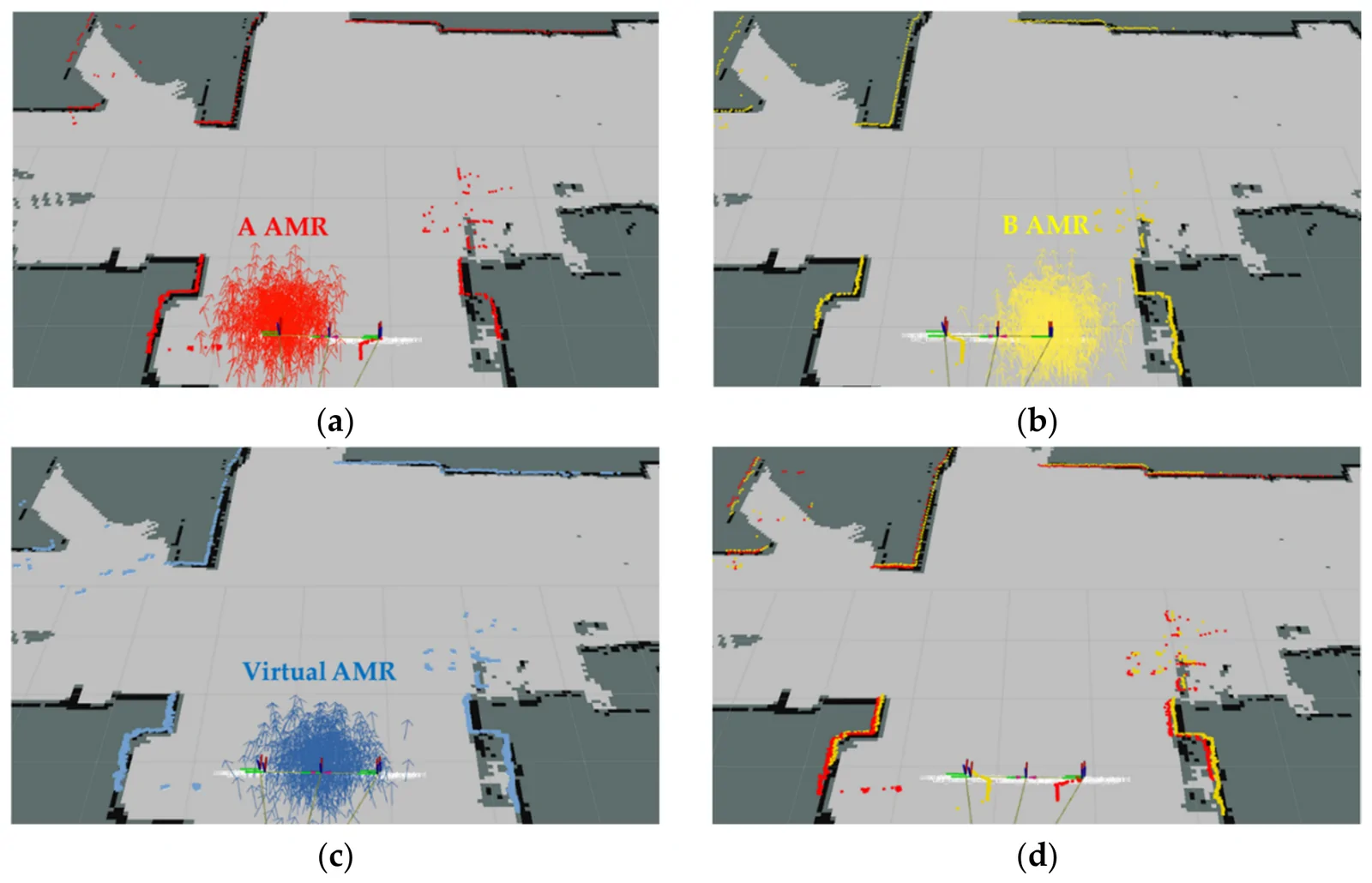
Environmental data observed by the two AMRs and the synthesized Virtual-AMR in the initial state. (**a**) LiDAR data from AMR A; (**b**) LiDAR data from AMR B; (**c**) Virtual-AMR LiDAR data; and (**d**) simultaneous LiDAR data from AMRs A and B.

Figure 13 compares the LiDAR observations of AMR A, AMR B, and the Virtual-AMR in the initial state. Because AMRs A and B occupy different positions, their fields of view are complementary. Figure 13d displays their raw scans simultaneously in the common map coordinate frame to illustrate the observation sources subsequently used for robot return identification, coordinate transformation, and virtual scan fusion.

**Figure 14:**
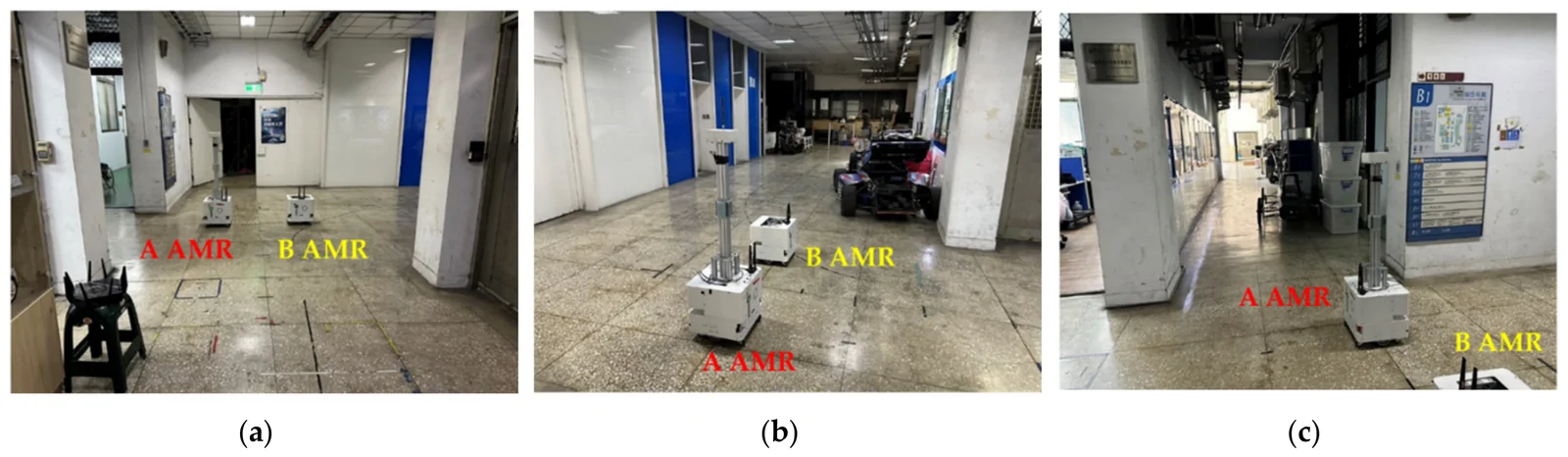
Geometry of the experimental area. (**a**) Front view; (**b**) right-side view; and (**c**) left-side view.

**Figure 15:**
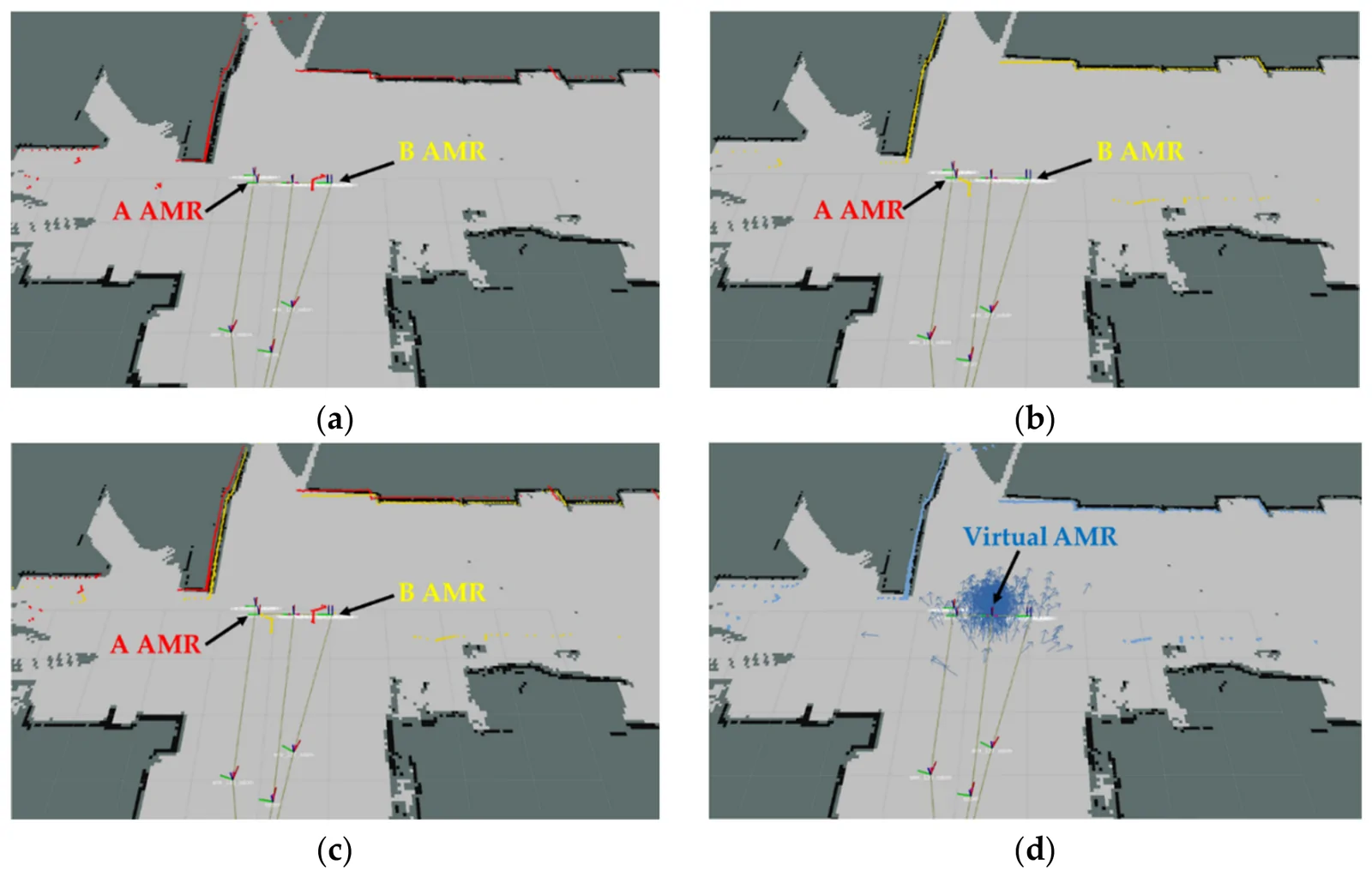
Environmental data observed by the two AMRs and the synthesized Virtual-AMR after motion. (**a**) LiDAR data from AMR A; (**b**) LiDAR data from AMR B; (**c**) combined LiDAR data from AMRs A and B; and (**d**) Virtual-AMR LiDAR data.

After the two AMRs have moved 4 m forward along a straight line, fewer identifiable static geometric features are available laterally, and the neighboring AMR further occludes some environmental returns, as shown in Figure 14 and Figure 15a,b. Each individual scan therefore contains both missing static measurements caused by occlusion and body returns from the other AMR. Following the procedure in Section 4.4, the system transforms valid measurements from both AMRs into a common frame, removes the returns identified as AMR bodies, and fuses the retained environmental points to construct the Virtual-AMR scan, as shown in Figure 15c,d. This analysis primarily verifies that the virtual scan supplements environmental information from complementary viewpoints. Its quantitative effect on localization error is evaluated in the subsequent experiments.

#### 5.2.2. Relative Angle Estimation and Computation Time

The KD-tree branch in Figure 3 was validated using RViz data. The initial full-angle search obtained the maximum overlap score at 1.8° and required 2253 ms (approximately 0.44 Hz). Subsequent local refined searches were centered on this estimate and required 150 ms (approximately 6.67 Hz) under the straight-line condition and 172 ms (approximately 5.81 Hz) under the 90° turning condition. The 2253 ms full-angle search was used only for initialization or recovery. After synchronization, preprocessing, tracking, virtual scan construction, and AMCL updating were included, the sum of the processing module times in Table 4 yielded complete end-to-end update rates of 4.08 Hz (straight line, 245 ms) and 3.46 Hz (90° turning, 289 ms). The normal processing rate is therefore lower than the raw 15 Hz LiDAR data rate. The 0.1° value is an angular-optimization increment of the algorithm, not the physical angular resolution of the TiM551.

**Figure 16:**
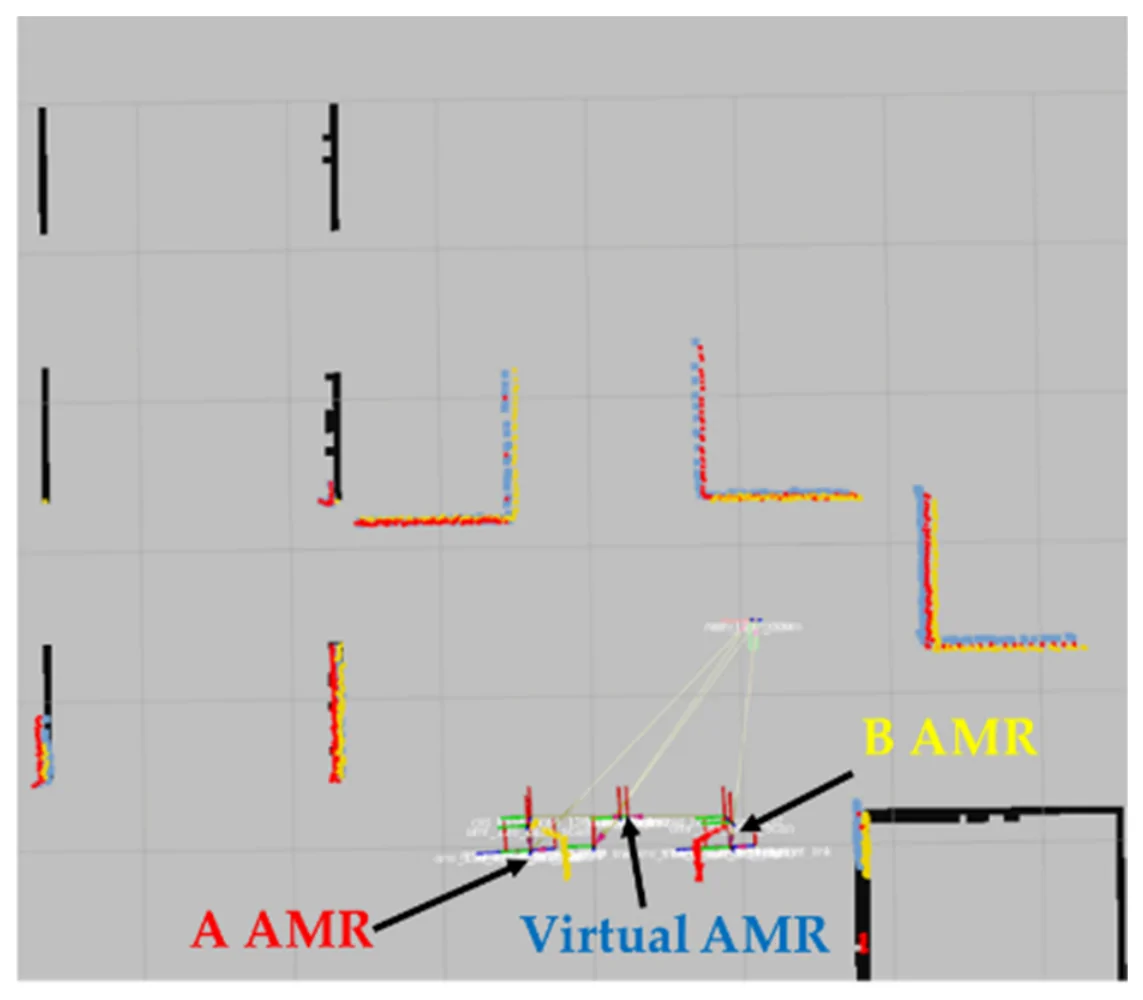
Distribution of AMR LiDAR scans.

**Figure 17:**
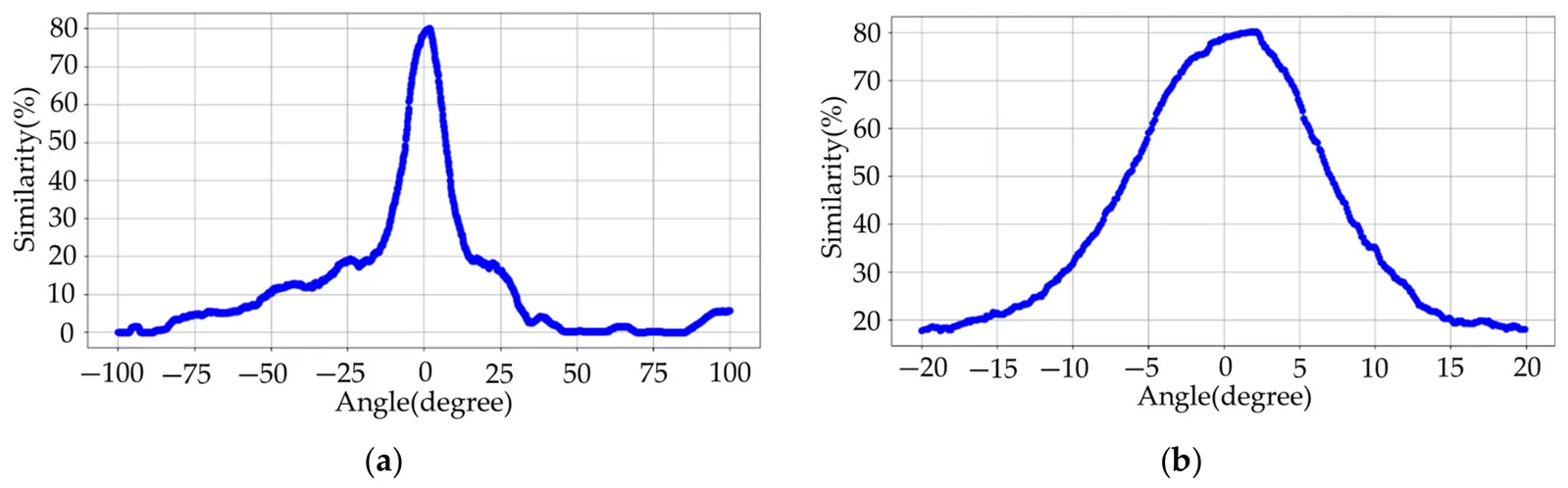
KD-tree angular search results. (**a**) Full-range angular search; (**b**) local refined angular search.

**Figure 18:**
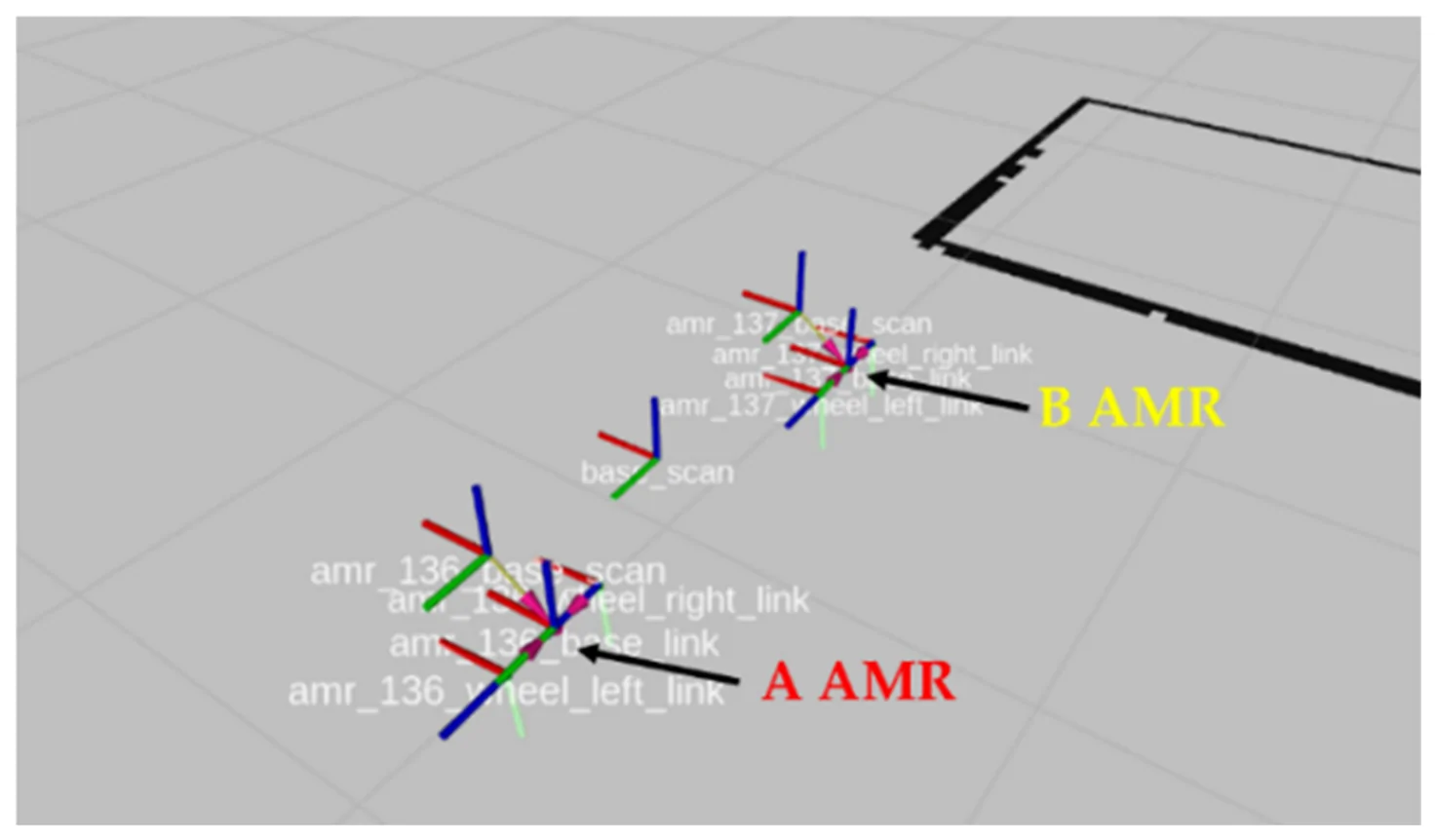
Relative coordinates of AMRs A and B.

Figure 16 and Figure 17 show the scan distributions and angular search results under mutual occlusion. Because the right-side field of view of AMR A is occluded by AMR B, the scan from AMR B is transformed over candidate angles and overlap-matched with the scan from AMR A. The initialization search reaches its highest matching score at 1.8°, after which refinement is centered on this angle. Once the relative heading has been estimated, the angle branch and the feature recognition/tracking branch jointly form the relative SE(2) pose and TF relationship shown in Figure 18. These results are then used for robot return masking and Virtual-AMR scan fusion as described in Section 4.4.

Table 5 additionally reports CPU utilization on the Intel Pentium G4400 platform. In normal refined-search mode, CPU utilization is 49–66% under the straight-line condition and 57–71% under the 90° turning condition. The corresponding end-to-end latencies are listed in Table 4. The full-angle search has a total latency of approximately 2.36–2.38 s and may reach 74–93% CPU utilization. It is therefore used only for initialization/recovery rather than during every scan in normal operation.

These latency and update-rate measurements characterize only the tested centralized dual-AMR configuration and the hardware listed in Table 3. They should not be interpreted as evidence of real-time scalability to larger robot teams.

#### 5.2.3. Sensitivity to KD-Tree Parameters

KD-tree relative angle estimation is primarily affected by the correspondence distance gate ε_d and the angular optimization increment Δ*θ*. The nominal settings are *ε_d_* = 3 mm and Δ*θ* = 0.1°. The 3 mm value is the geometric consistency gate for the post-filtered point sets, while 0.1° is the optimization step of the search algorithm. Neither value represents the nominal ranging accuracy or physical angular resolution of the TiM551. Parameter stability was evaluated using a one-factor-at-a-time analysis: *ε_d_* was varied while Δ*θ* was fixed at 0.1°, and Δ*θ* was varied while *ε_d_* was fixed at 3 mm.

All sensitivity tests used the same dual-AMR configuration, localization parameters, and motion conditions. The failure rate, two-dimensional position RMSE, heading RMSE, KD-tree plus refined-search time, and end-to-end latency in Table 6 and Table 7 are measured sensitivity test results. Failure rate is defined as the proportion of test updates for which the KD-tree branch failed to produce a valid relative heading estimate according to the matching conditions in Section 4.3.

Table 6 shows a clear tradeoff in the distance gate. Increasing *ε_d_* from 1 to 3 mm reduces the failure rate from 11.7% to 2.0% and the two-dimensional position RMSE from 5.2 to 3.5 cm. Further relaxing the gate to 7 mm increases the failure rate to 8.3%, raises the RMSE to 5.0 cm, and increases the search time to 205 ms. Thus, *ε_d_* = 3 mm provides the best balance between matching stability and computational demand in the present dual-AMR experiments.

Table 7 shows that Δ*θ* = 0.05° reduces heading RMSE from 0.16° to 0.12°, but increases the KD-tree plus refined-search time from 150 to 310 ms. Conversely, increments of 0.5° and 1.0° reduce computation time but raise the failure rate to 7.5% and 15.0%, respectively. The proposed method therefore retains *ε_d_* = 3 mm and Δ*θ* = 0.1° as the nominal settings. This conclusion is limited to the two AMRs and the test site conditions evaluated in this study.

### 5.3. Straight-Line Localization and Stability Analysis

This section analyzes four signal categories obtained in the straight-line experiments: AMCL tracking error, particle cloud dispersion, Virtual-AMR center-position consistency, and odometry-related internal consistency. Finally, the branch-wise ablation directly compares the T1–T4 configurations to assess how the two main branches—relative angle estimation and feature recognition/tracking—affect localization performance under the straight-line condition.

#### 5.3.1. AMCL Localization Error and Stability

**Figure 19:**
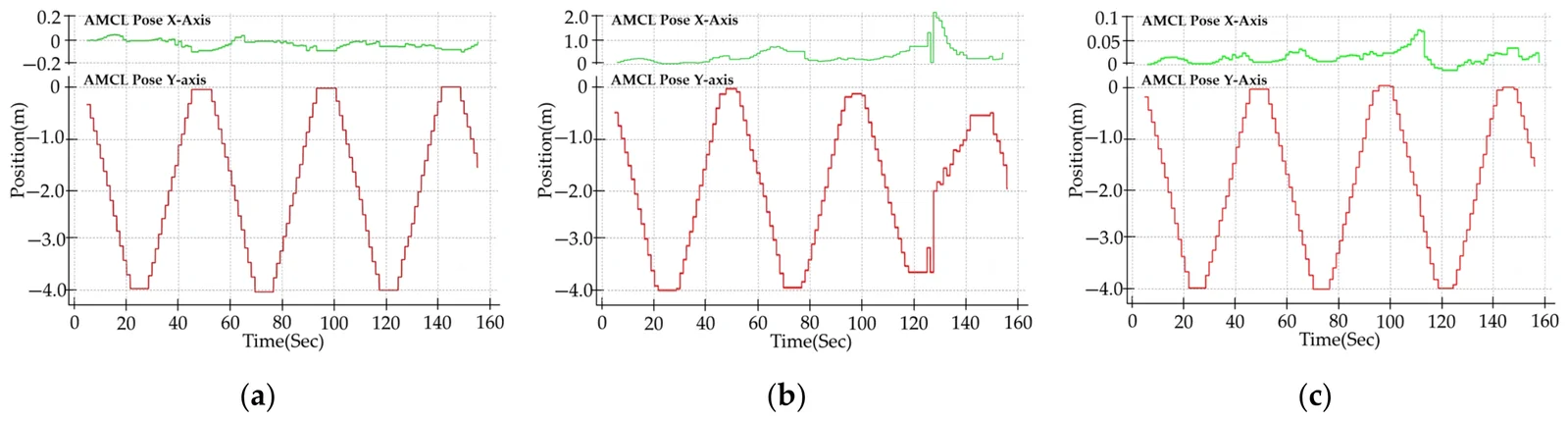
AMCL position estimates. (**a**) AMR A; (**b**) AMR B; and (**c**) Virtual-AMR.

**Figure 20:**
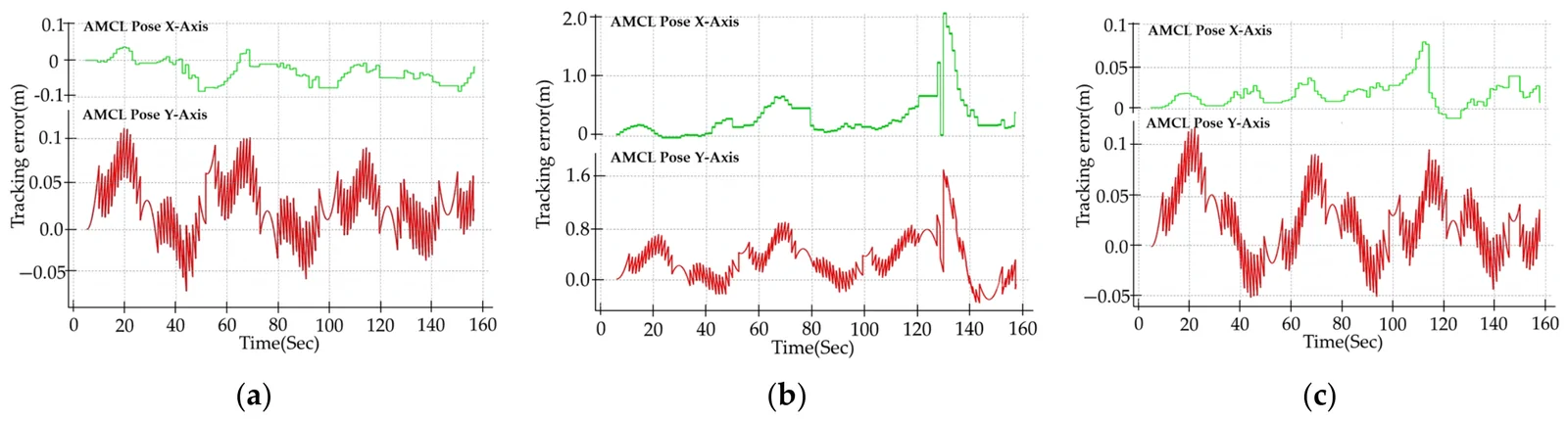
AMCL tracking errors. (**a**) AMR A; (**b**) AMR B; and (**c**) Virtual-AMR.

Figure 19 shows the amcl_pose results from a representative straight-line experiment. Because AMR A observes a more complete set of static map features, its estimate remains stable. After AMR B becomes occluded by AMR A, it exhibits pronounced localization divergence beginning at approximately 130 s. With the complete Virtual-AMR pipeline, the same type of divergence does not occur in Figure 19c. Figure 20 further compares each localization output with the reference trajectory to show the change in tracking error before and after mutual occlusion.

Table 8 quantifies these differences. The two-dimensional RMSE values for AMR A, AMR B, and the Virtual-AMR are 0.0479, 0.4995, and 0.0426 m, respectively, and their P95 errors are 0.1006, 1.0326, and 0.0949 m, respectively. Under the straight-line occlusion condition evaluated here, the Virtual-AMR output substantially reduces the tracking error of the occluded AMR B while maintaining an error range comparable to that of AMR A, which does not exhibit marked divergence.

#### 5.3.2. Particle Cloud Dispersion Analysis

**Figure 21:**
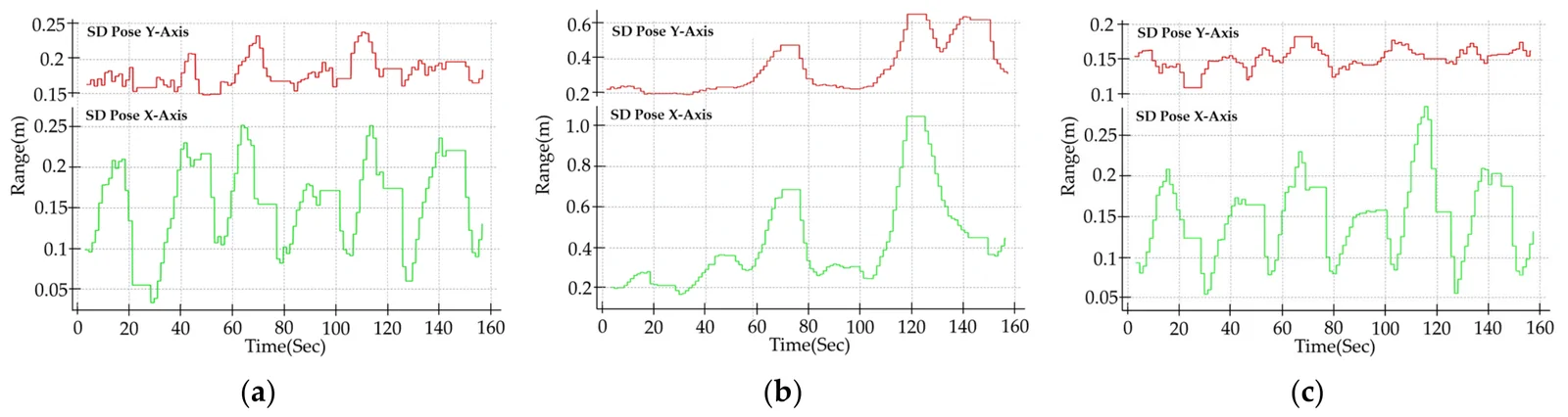
Particle cloud distributions. (**a**) AMR A; (**b**) AMR B; and (**c**) Virtual-AMR.

Figure 21 uses the spatial dispersion of the particle cloud to examine changes in uncertainty of the AMCL posterior. When AMR B is occluded and its localization diverges, its particle cloud is markedly more dispersed than those of AMR A and the Virtual-AMR. The Virtual-AMR maintains a more concentrated posterior distribution.

Table 9 summarizes the particle cloud dispersion statistics. The two-dimensional RMSE values for AMR A, AMR B, and the Virtual-AMR are 0.3080, 0.6267, and 0.2623 m, respectively. AMR B has the greatest dispersion, consistent with the increased particle weight uncertainty during its divergence in Figure 19. The Virtual-AMR particle cloud remains comparatively concentrated.

#### 5.3.3. Virtual-AMR Center-Position Consistency

**Figure 22:**
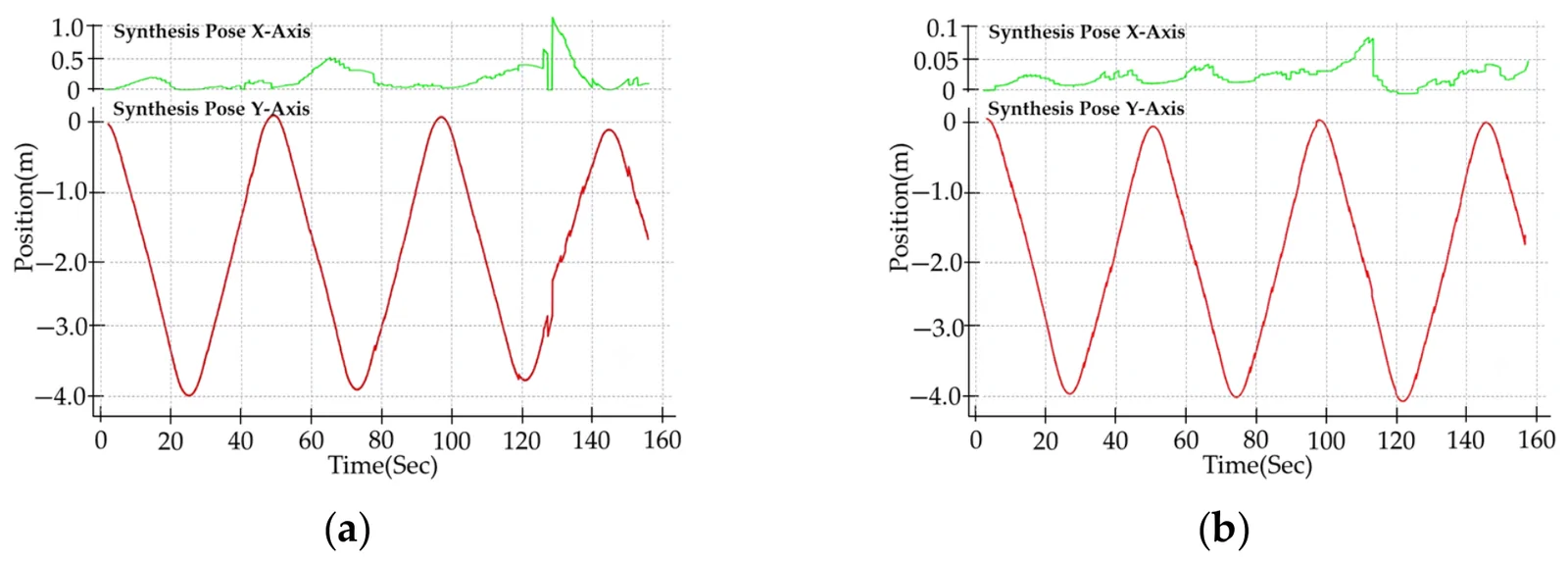
Central-position estimates. (**a**) A/B spatial midpoint baseline; (**b**) Virtual-AMR.

**Figure 23:**
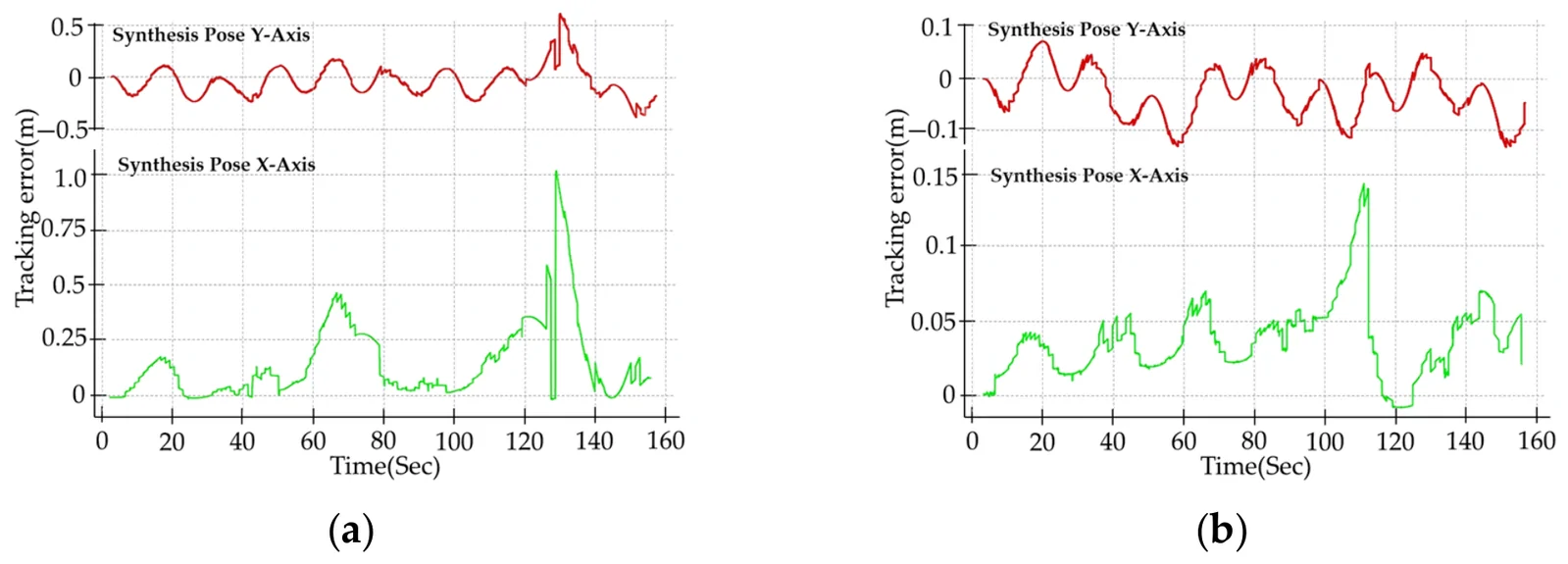
Central-position estimation errors. (**a**) A/B spatial midpoint baseline; (**b**) Virtual-AMR.

Figure 22a directly forms a spatial midpoint from the AMCL base_footprint poses of AMRs A and B, providing a central-position baseline that does not use either processing branch of the proposed method. When AMR B diverges, this direct midpoint is also affected by its erroneous pose. Figure 22b shows the Virtual-AMR position obtained using the proposed relative-position/heading estimation and virtual scan construction. Figure 23 compares the errors of these two estimates relative to the reference trajectory.

Table 10 shows that the direct A/B spatial midpoint baseline and the Virtual-AMR have two-dimensional RMSE values of 0.2426 and 0.0597 m, respectively, and P95 errors of 0.4765 and 0.1147 m, respectively. Thus, when AMR B drifts under occlusion, simply averaging the two AMCL poses propagates the error, whereas the complete Virtual-AMR pipeline maintains a smaller central-position tracking error.

#### 5.3.4. Odometry-Related Internal Consistency

**Figure 24:**
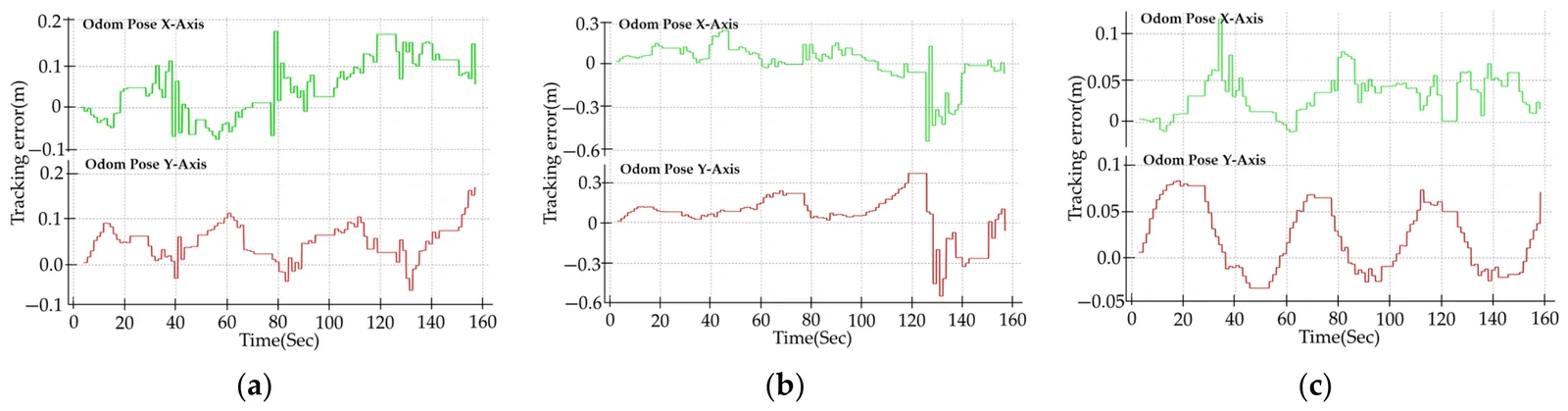
Odometry-related data. (**a**) AMR A; (**b**) AMR B; and (**c**) Virtual-AMR.

Figure 24 compares odometry-related displacement signals with the aligned reference trajectory to examine internal motion consistency across configurations. The error of AMR A remains comparatively small. AMR B shows greater inconsistency after occlusion causes AMCL drift, while the synthesized Virtual-AMR result remains more concentrated.

Table 11 shows two-dimensional RMSE values of 0.0863, 0.2469, and 0.0475 m for AMR A, AMR B, and the Virtual-AMR, respectively. AMR B exhibits greater internal inconsistency, a trend consistent with its AMCL tracking error and particle cloud dispersion.

Taken together, Table 8, Table 9, Table 10 and Table 11 show that the AMCL tracking error and particle cloud dispersion of AMR B increase markedly under mutual occlusion in the straight-line experiment, whereas the Virtual-AMR results maintain smaller tracking errors and a more concentrated posterior distribution. The following branch-wise ablation provides the primary direct quantitative comparison of the two main processing branches.

#### 5.3.5. Branch-Wise Ablation

To directly evaluate the contributions of the two main branches in Figure 3, Table 12 summarizes the complete T1–T4 settings and their relative-position/heading sources. T1 uses standard individual-scan AMCL/TF. For central-position comparison with T2–T4, an evaluation-only direct spatial midpoint is formed from the A/B AMCL/TF outputs; it does not use the relative angle branch, feature recognition/tracking branch, robot return masking, or Virtual-AMR fused scan. T2 activates only the relative angle estimation branch, T3 activates only the feature recognition/tracking branch, and T4 activates both branches and constructs the complete Virtual-AMR scan. All four configurations use the same two AMRs, initial spacing, and motion parameters, and each was evaluated in five independent repeated trials. As shown in Table 13, the two-dimensional RMSE values for T1, T2, T3, and T4 under the straight-line condition are 0.2426, 0.1607, 0.1227, and 0.0426 m, respectively. Relative to T1, T4 reduces the two-dimensional RMSE by approximately 82.4% and also outperforms both single-branch configurations, indicating complementary effects of the position and heading branches. This ablation quantifies only the two principal branches and their integration; it does not claim to isolate the individual contribution of every submodule within each branch.

### 5.4. Dual-AMR Turning Validation

**Figure 25:**
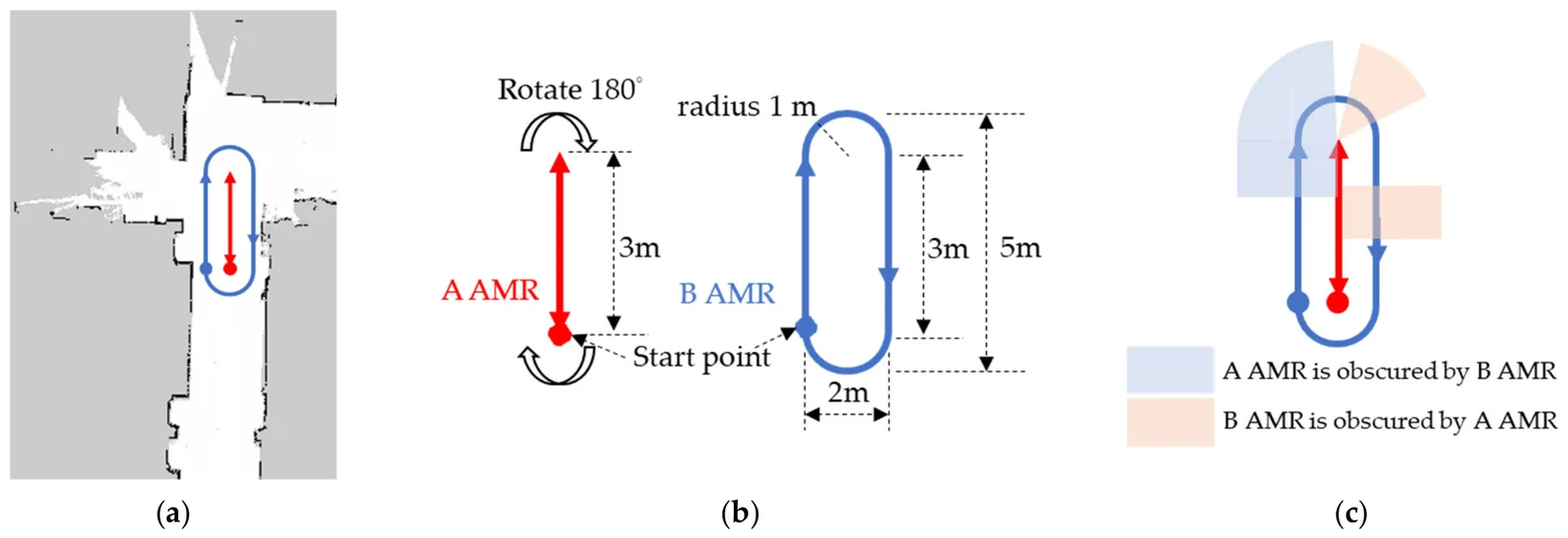
Dual-AMR turning path: (**a**) path location in the map; (**b**) trajectories of AMRs A and B; and (**c**) mutual occlusion region of AMRs A and B.

**Figure 26:**
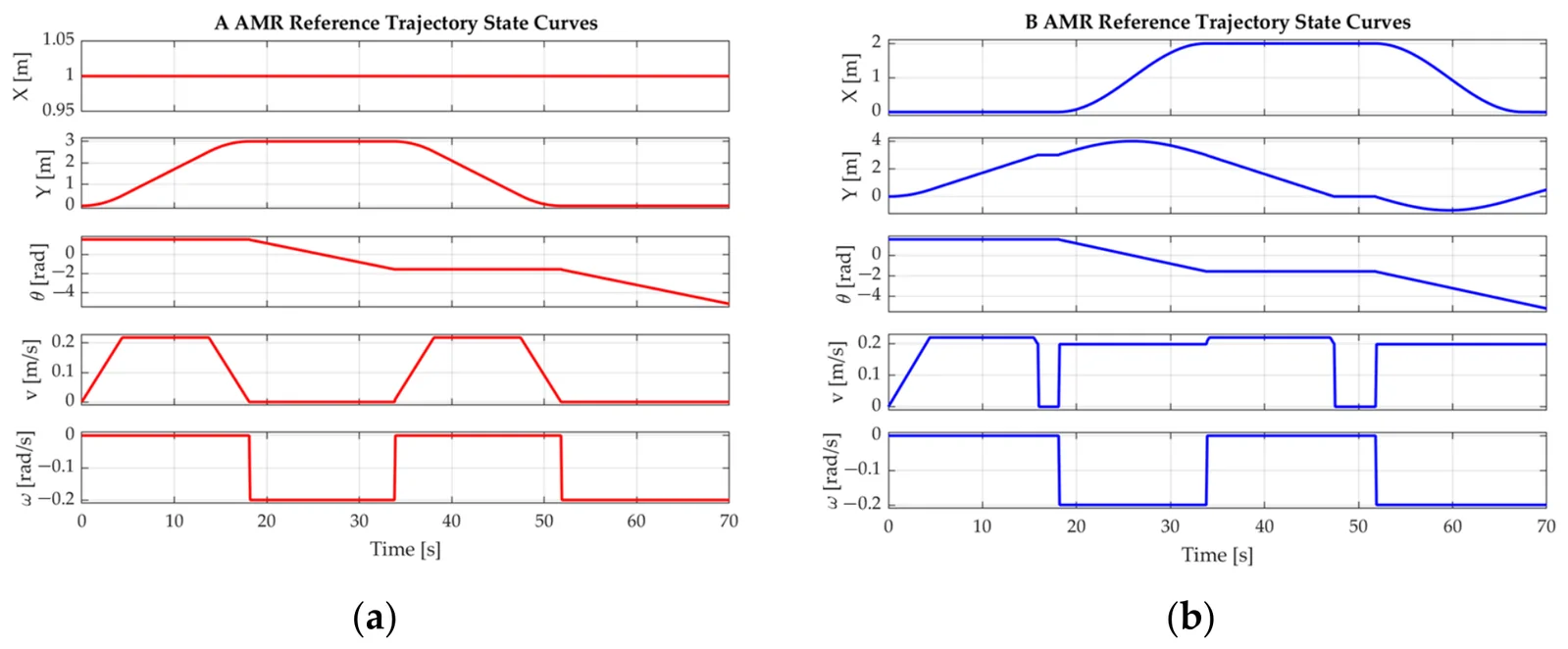
Positions and velocities of the dual-AMRs: (**a**) AMR A; (**b**) AMR B.

This section extends the same T1–T4 configurations to a dual-AMR turning trajectory to evaluate rapid relative-heading changes, position exchange, and mutual occlusion during turns. Figure 25a shows the path location in the experimental map, Figure 25b presents the planned trajectories of AMRs A and B, and the occlusion region in Figure 25c is jointly determined by the relative geometry of the two AMRs and the visible features of the test area. Figure 26 presents the displacement and velocity settings for the two AMRs. All configurations use the same motion conditions to ensure consistency in the branch-wise comparison.

**Figure 27:**
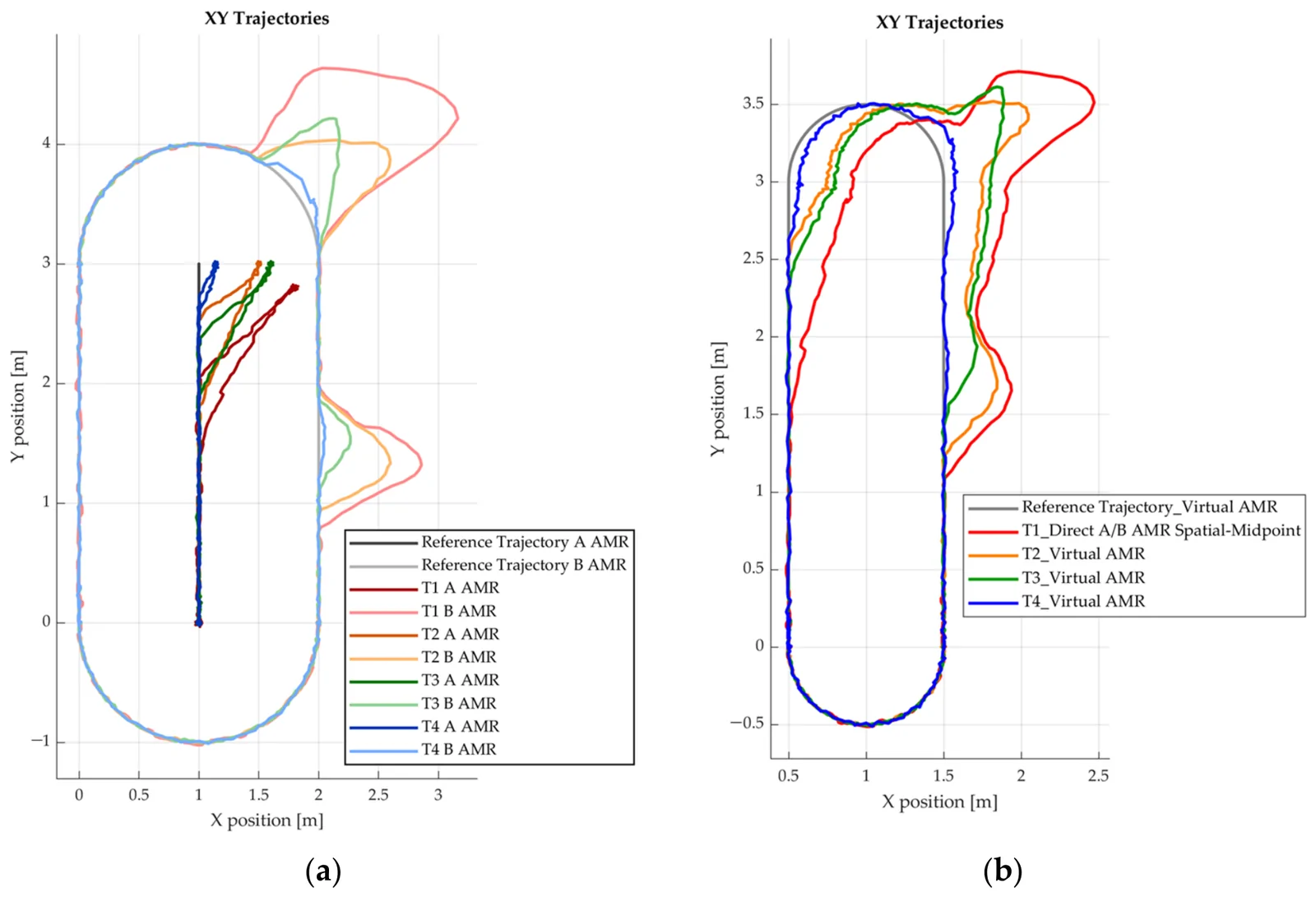
Dual-AMR trajectories in the experiments: (**a**) trajectories of AMRs A and B; (**b**) estimated central path.

Each T1–T4 configuration in Table 12 was evaluated in five independent repeated trials, with 30 laps per trial while localization and control data were recorded. Figure 27 shows the 20th lap of one representative experiment: Figure 27a presents the estimated trajectories of AMRs A and B, and Figure 27b presents the corresponding central-position estimate. T1 is the direct AMCL/TF spatial midpoint baseline, while T2–T4 represent the single-branch and complete Virtual-AMR configurations.

**Figure 28:**
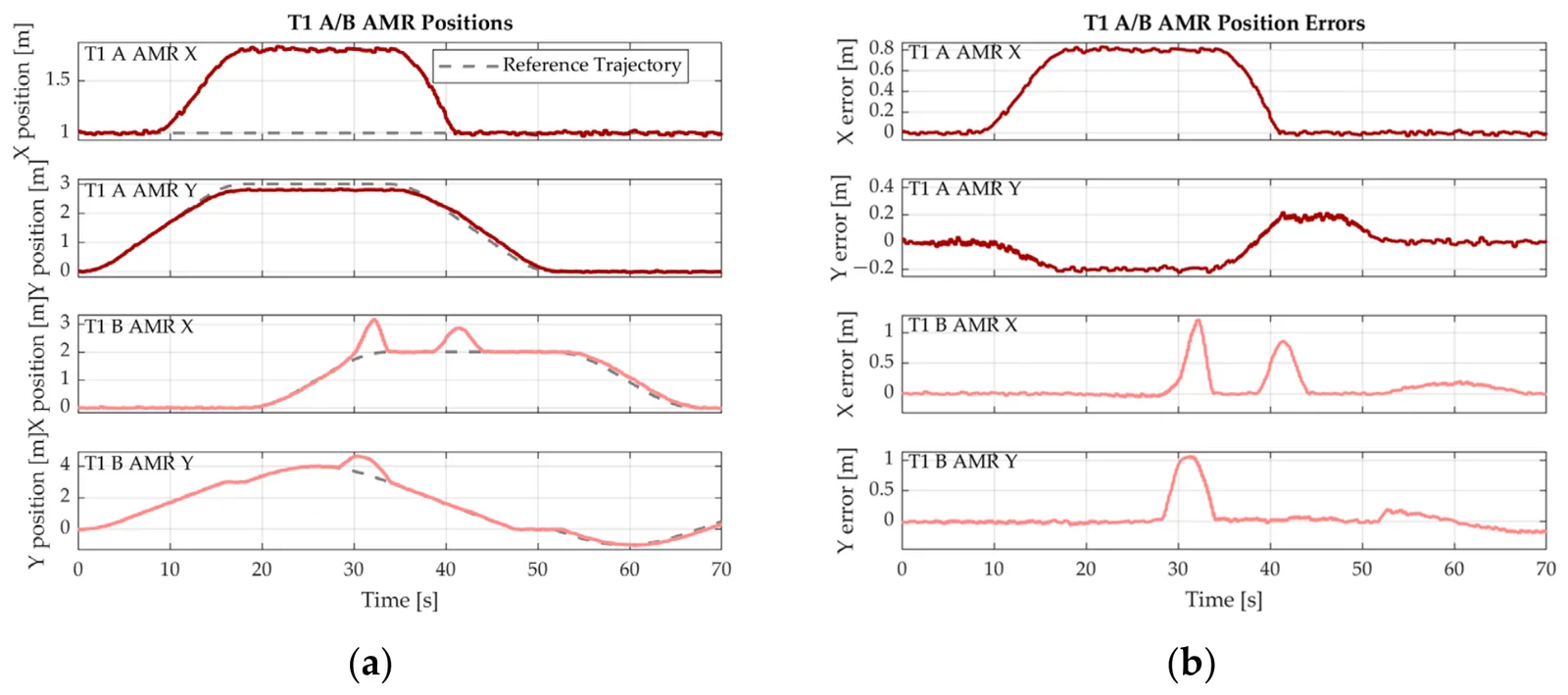
Dual-AMR results under T1: (**a**) AMR trajectories; (**b**) position errors.

In the representative T1 trajectory, AMR A begins to drift after reaching approximately Y = 2 m because its left side is occluded by AMR B and relatively few usable static features are available in the remaining directions. Its maximum x-direction deviation is approximately 0.8 m. During the turn, AMR B enters a more severe occlusion region at approximately 120° and exhibits a deviation of approximately 1.2 m. It subsequently returns near the planned path after recovering sufficient environmental features. Another deviation of approximately 0.8 m is observed on the return segment. Figure 28 shows the errors of this representative T1 trajectory relative to the reference trajectory. This single-lap description identifies where occlusion occurs; the overall statistics are based on data from the five repeated trials.

**Figure 29:**
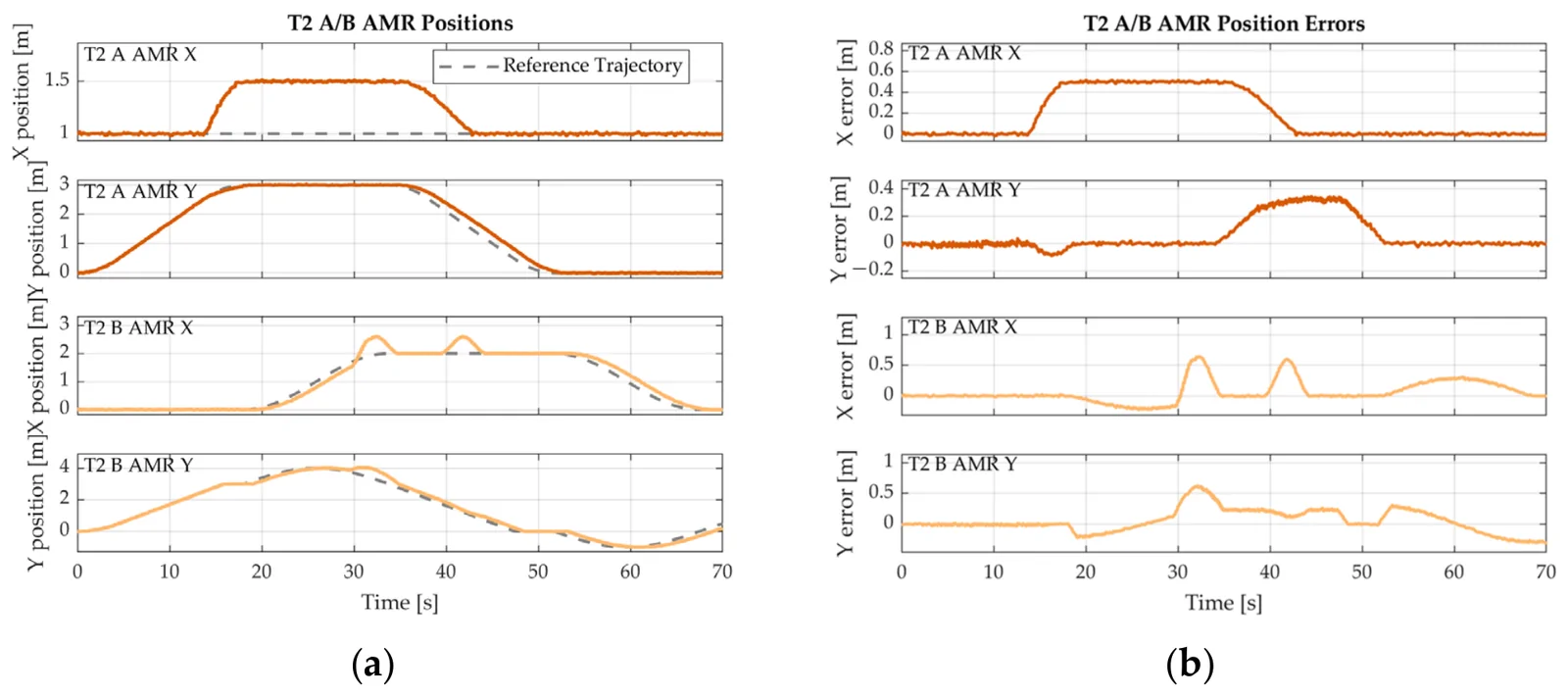
Dual-AMR results under T2: (**a**) AMR trajectories; (**b**) position errors.

**Figure 30:**
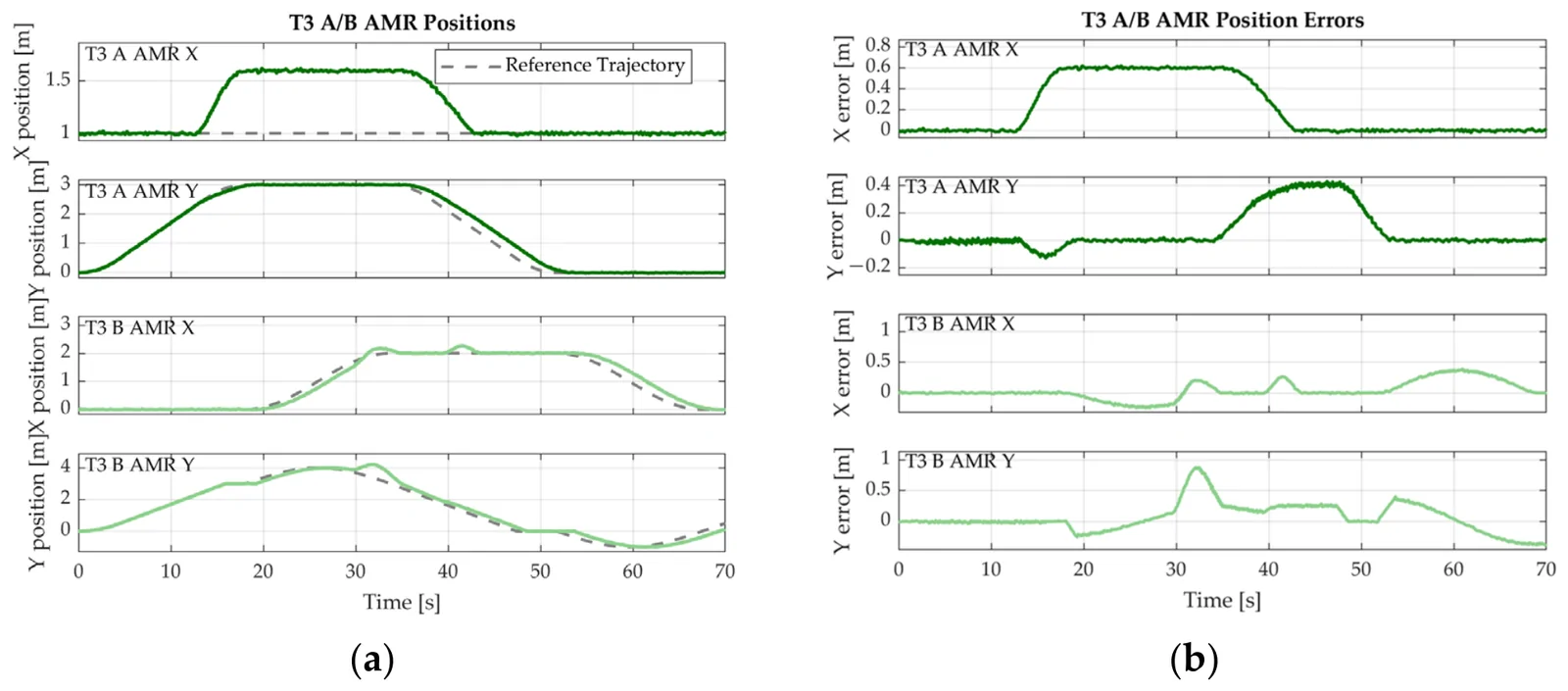
Dual-AMR results under T3: (**a**) AMR trajectories; (**b**) position errors.

**Figure 31:**
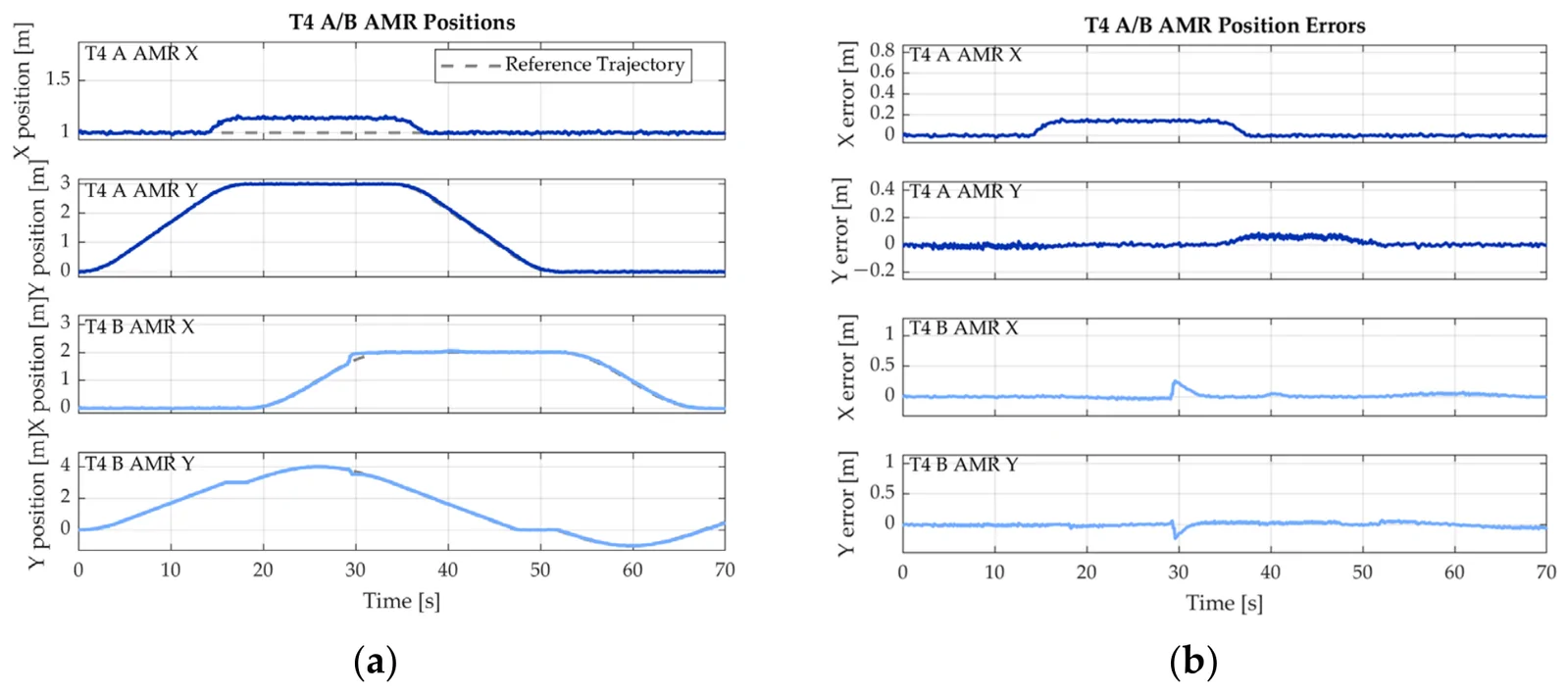
Dual-AMR results under T4: (**a**) AMR trajectories; (**b**) position errors.

Figure 29, Figure 30 and Figure 31 show representative results for the three improved configurations. T2 uses only the relative angle branch and reduces part of the heading drift, but its position estimate remains affected by standard AMCL/TF error. T3 uses only the feature recognition/tracking branch and improves relative position, but lacks KD-tree heading correction during rapid turning. T4 uses both branches. In the representative trajectory, the maximum path deviations of AMRs A and B decrease to approximately 0.16 and 0.35 m, respectively, indicating that the joint use of position and heading information further suppresses drift during occlusion.

**Figure 32:**
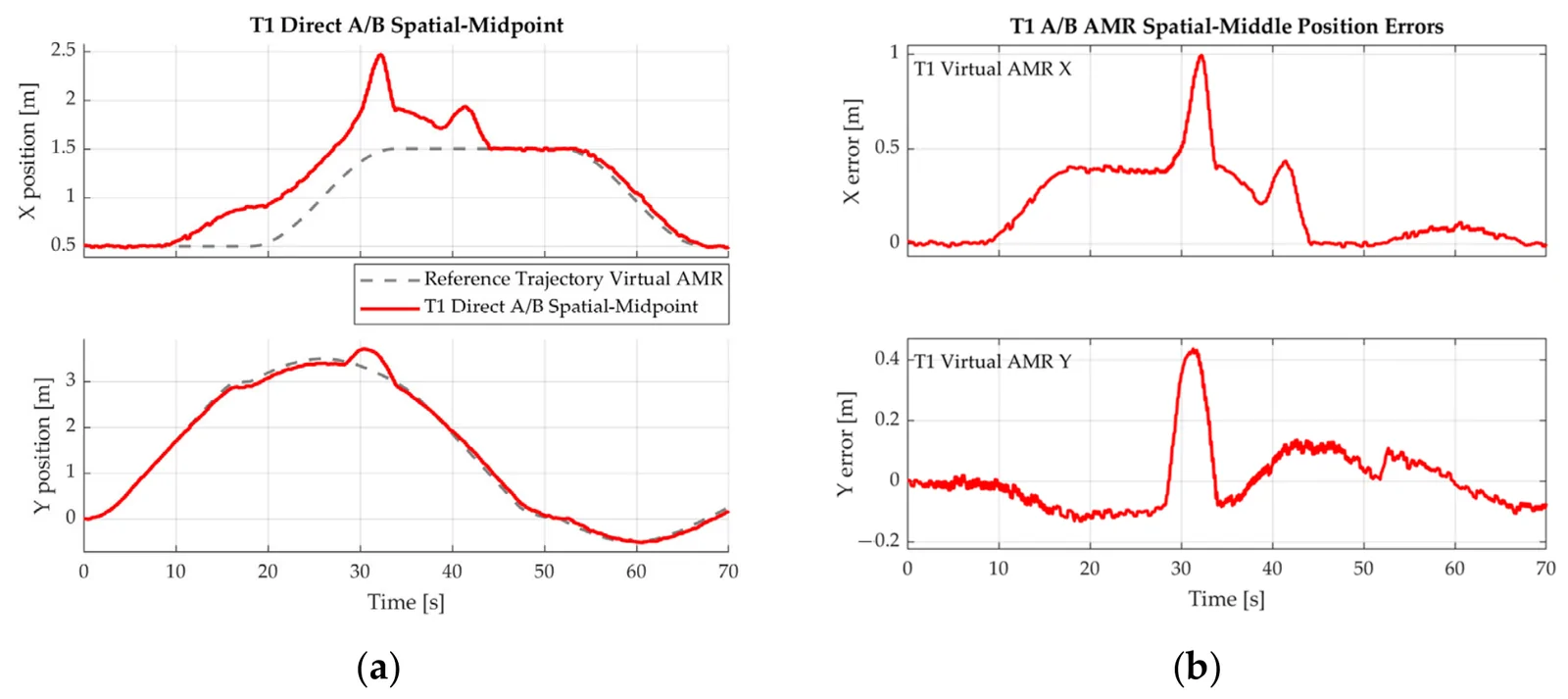
A/B spatial midpoint baseline under T1: (**a**) estimated trajectory; (**b**) position error.

**Figure 33:**
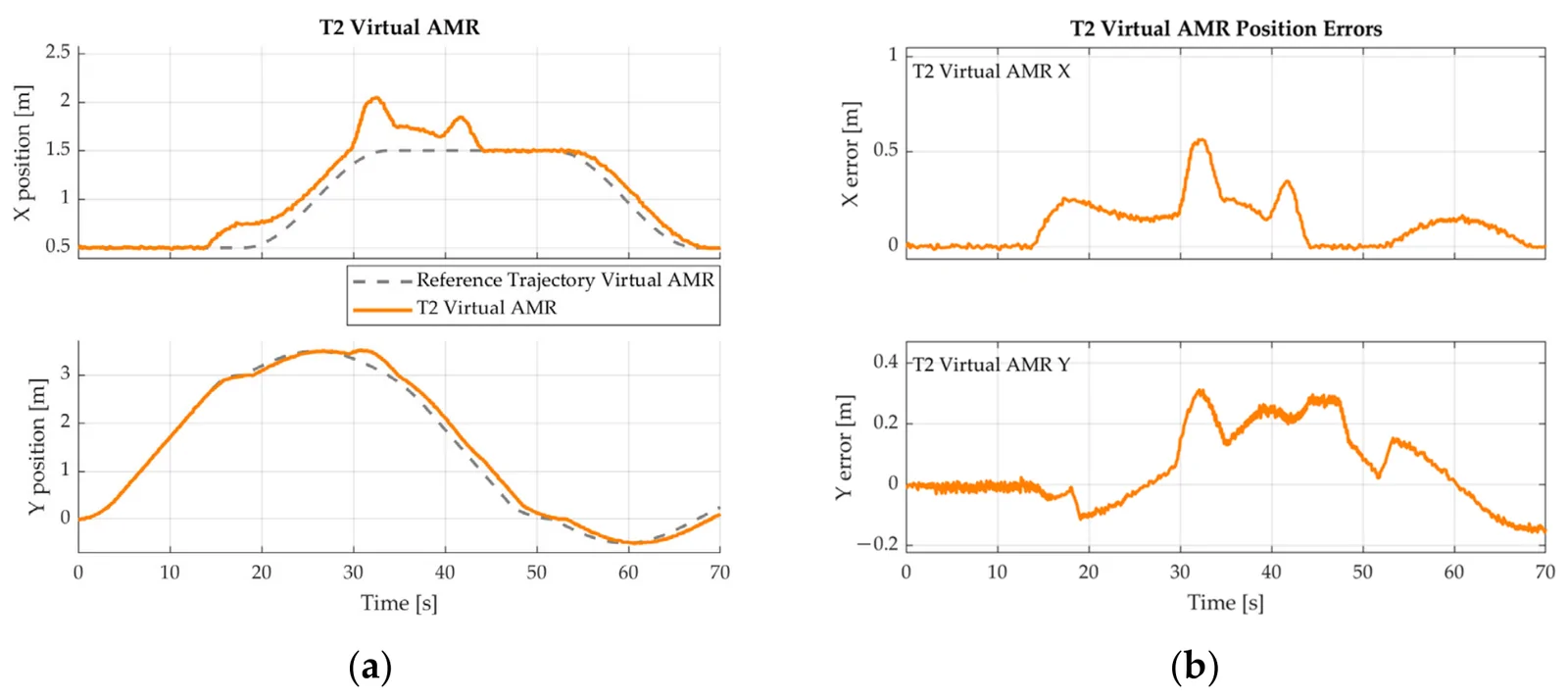
Central-position estimate under T2: (**a**) estimated trajectory; (**b**) position error.

**Figure 34:**
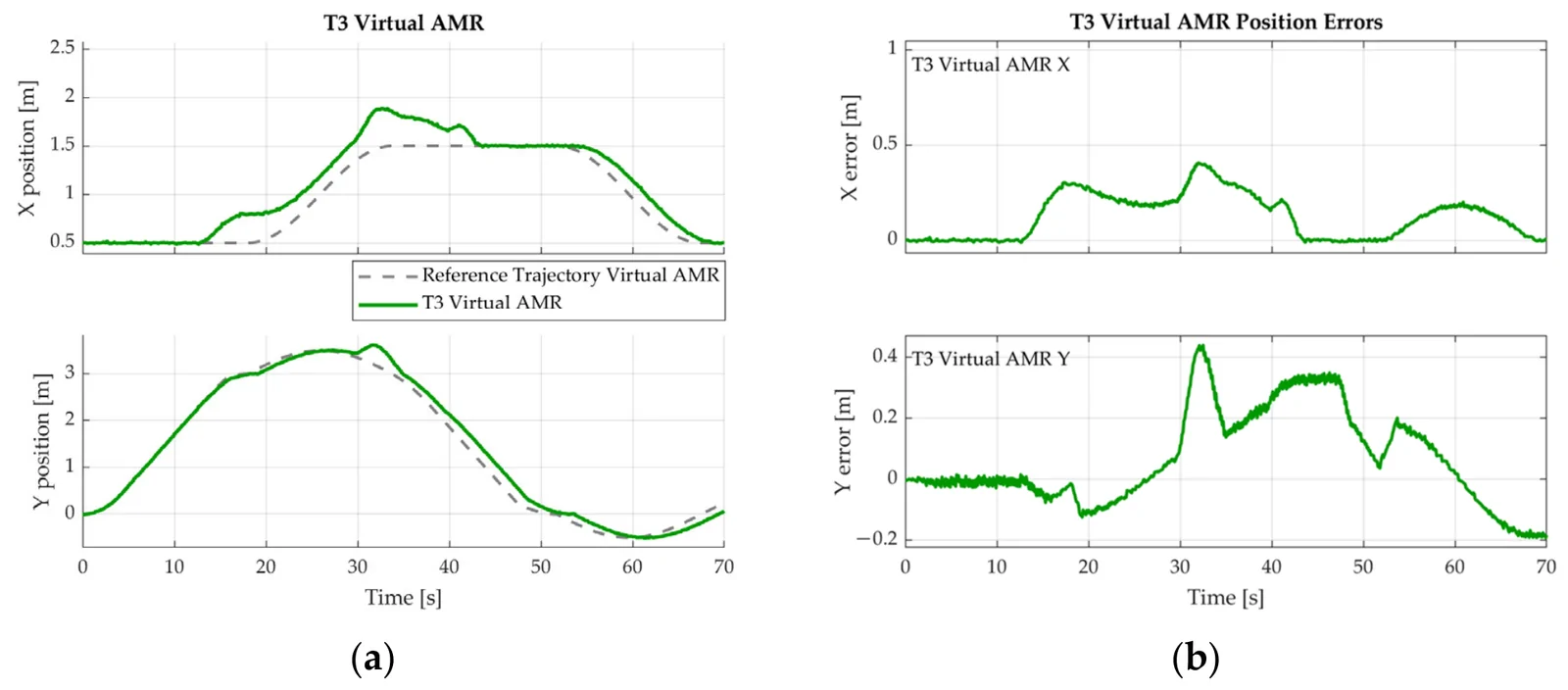
Central-position estimate under T3: (**a**) estimated trajectory; (**b**) position error.

**Figure 35:**
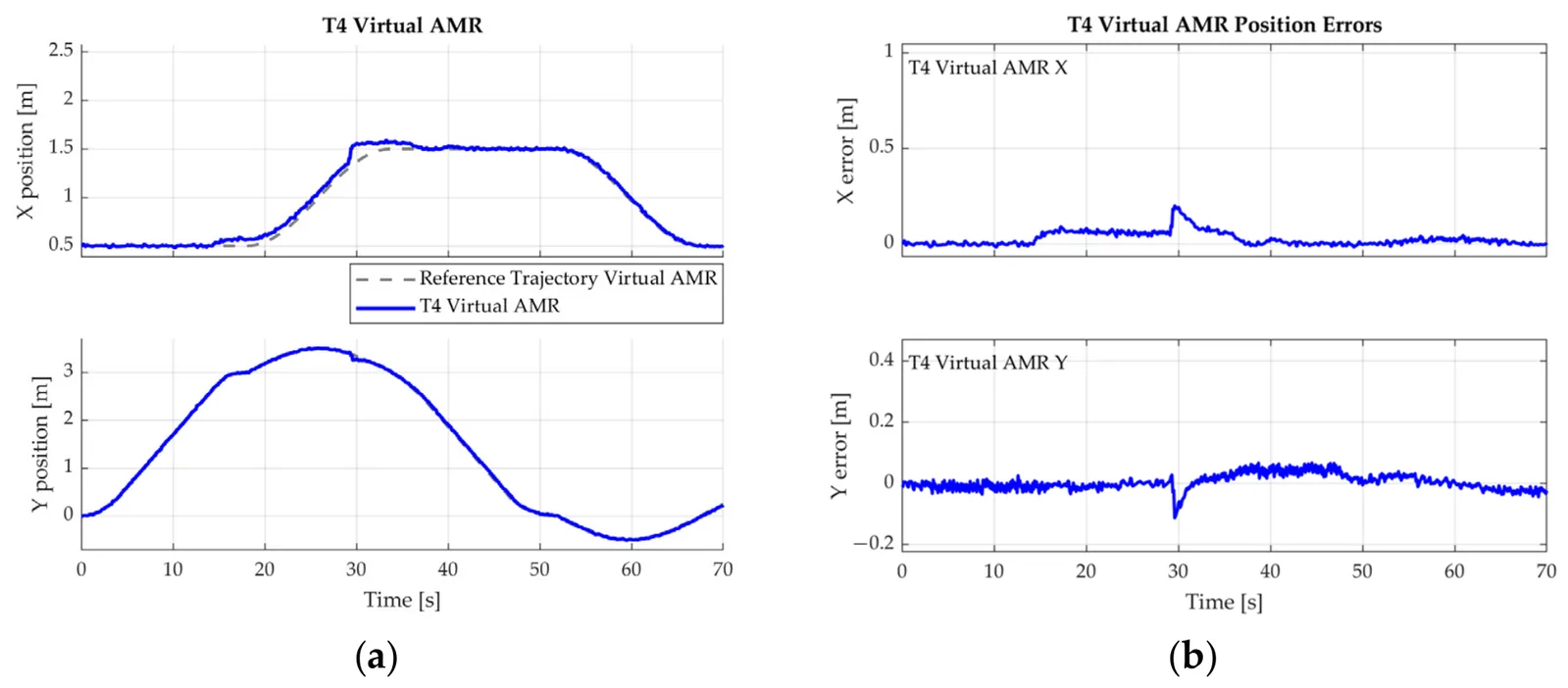
Complete Virtual-AMR localization under T4: (**a**) estimated trajectory; (**b**) position error.

Figure 32, Figure 33, Figure 34 and Figure 35 compare the central-position estimates of T1–T4. T1 is a direct spatial midpoint baseline formed from the individual A/B AMCL/TF outputs rather than from an average of the LiDAR data. Consequently, divergence of either physical AMR directly affects the central estimate. T2 and T3 each add one processing branch and reduce part of the deviation, while T4 integrates relative position and relative heading.

In Table 14, the two-dimensional RMSE values for T1, T2, T3, and T4 are 0.3088, 0.2208, 0.2411, and 0.0521 m, respectively. Relative to T1, T4 reduces two-dimensional RMSE by approximately 83.1% and clearly outperforms both single-branch configurations. The two-dimensional RMSE of T4 under the turning condition (0.0521 m) is higher than that under the straight-line condition (0.0426 m), indicating that relative motion and changes in visible geometry during turning increase estimation difficulty. Future work may investigate adaptive *w_i_* weights based on localization quality; however, that strategy was not evaluated in this study and is therefore not reported as a validated result.

For each T1–T4 configuration under each trajectory condition, the 2D RMSE was calculated separately for each of the five trials and summarized using the arithmetic mean and sample standard deviation, so each trial contributed equally regardless of the number of valid samples. Table 15 and Table 16 report the individual trial values and mean ± sample standard deviation for the straight-line and turning experiments, respectively, whereas the pooled MAE, RMSE, E_max, E_med, and E_P95 values in Table 8, Table 13 and Table 14 are retained as descriptive statistics of the complete error sample distributions. Under the straight-line condition, the trial-level mean ± sample standard deviation values for T1–T4 were 0.2401 ± 0.0365, 0.1595 ± 0.0215, 0.1210 ± 0.0187, and 0.0412 ± 0.0083 m, respectively. Under the turning condition, the corresponding values were 0.3047 ± 0.0563, 0.2191 ± 0.0395, 0.2401 ± 0.0417, and 0.0511 ± 0.0104 m. T4 therefore achieved the lowest trial-level mean 2D RMSE under both trajectory conditions.

## 6. Conclusions

Mutual occlusion between neighboring AMRs can reduce observable static map features and introduce robot body returns, causing AMCL drift or divergence. This study developed a Virtual-AMR LiDAR fusion method that integrates contour recognition and ROI tracking, linear Kalman filtering, KD-tree relative-heading estimation, robot return masking, and SE(2) transformation to fuse usable environmental measurements from two AMRs before AMCL.

The method was evaluated in a 3.1 m × 10 m indoor area using controlled straight-line and turning trajectories. Each T1–T4 configuration was tested in five independent trials under both trajectory conditions. The 2D RMSE was calculated separately for each trial and summarized as the mean ± sample standard deviation, while pooled valid samples were retained for the detailed error distribution statistics. Relative to the internal reference trajectory, the complete T4 method achieved trial-level 2D RMSE values of 0.0412 ± 0.0083 m for the straight-line trajectory and 0.0511 ± 0.0104 m for the turning trajectory, outperforming the baseline and both single-branch configurations under both conditions. The measured end-to-end latency ranged from 245 to 289 ms, equivalent to update rates from 4.08 to 3.46 Hz. The full-angle search was used only for initialization or recovery.

These findings apply to the dual-AMR configuration evaluated in this study. To isolate the effects of mutual occlusion, the AMR platform, test site, initial spacing, and control parameters were fixed, and an internal reference trajectory was used to evaluate the relative performance and localization stability of each configuration. Future work will incorporate an external reference measurement system and further evaluate different inter-AMR spacings and configurations, occlusion levels, crossing paths, dynamic obstacles, and larger robot teams, while analyzing the computational and communication loads associated with increasing the number of robots. Quality-adaptive fusion and faster initialization and recovery strategies will also be investigated.

## Figures and Tables

**Table 1:** Comparison of the proposed method with representative cooperative localization and multi-robot SLAM approaches.

Approach	Shared Information	Primary Estimator Target	Difference from This Study
Distributed relative localization [4]	Inter-robot range/bearing or relative pose	Relative team configuration	Does not specifically reconstruct a virtual environmental scan for AMCL occlusion recovery
Resource-aware collaborative MCL [5]	Compressed particle belief distributions	Global localization belief	Fuses beliefs after robot detection rather than fusing masked LiDAR measurements before AMCL
Distributed multi-robot SLAM [6]	Descriptors, submaps, and pose graph constraints	Joint map and trajectory	Addresses collaborative mapping and loop closure, not the local mutual occlusion mechanism studied here
Proposed Virtual-AMR method	Timestamped 2D scans and inter-robot SE(2) transforms	Virtual measurement supplied to unchanged AMCL	Centralized measurement reconstruction for a known map; current validation is limited to two AMRs

**Table 2:** Algorithm and reproducibility parameters.

Parameter	Value
LiDAR point count, *ID_total_*	655
Median filter window	11 points
Smoothing index range, range	10 (11 inclusive samples)
Line fit neighborhood, *N*	10 points
KD-tree neighbor width	±10 indices
Post-filter distance gate, *ε_d_*	3 mm
Angular optimization increment	0.1°
Kalman sampling interval, Δ*t*	0.1 s
Kalman process covariance, **Q**	$Q=\left[\begin{array}{cccc} 6.25\times 10^{-8} & 0 & 1.25\times 10^{-6} & 0\\ 0 & 6.25\times 10^{-8} & 0 & 1.25\times 10^{-6}\\ 1.25\times 10^{-6} & 0 & 2.50\times 10^{-5} & 0\\ 0 & 1.25\times 10^{-6} & 0 & 2.50\times 10^{-5} \end{array}\right]$
Kalman measurement covariance, **R**	$R=\mathrm{diag}\left(4.0\times 10^{-4},4.0\times 10^{-4}\right)$
Kalman initial covariance, **P**_0_	$P_{0}=\mathrm{diag}\left(2.5\times 10^{-3},2.5\times 10^{-3},1.0\times 10^{-2},1.0\times 10^{-2}\right)$
ROI constants (*h_x_*_,0_, *h_y_*_,0_, *γ*)	0.25 m, 0.25 m, 2.0
Maximum inter-scan timestamp difference, Δ*t_sync,max_*	50 ms
Maximum observation age, *τ_age,max_*	100 ms
Normal E2E latency threshold, *T*_E2E,max_	350 ms
Consecutive invalid/missing synchronized pairs, *N*_miss,max_	3 frames

**Table 3:** AMR platform and communication specifications.

AMR Configuration	Specification
Operation system	Ubuntu 20.04.6 LTS 5.15.0-88-generic
ROS Middleware	ROS 2 Foxy Fitzroy
Navigation Stack	Navigation2 v0.4.7
Localization	nav2_amcl v0.4.7
CPU	Intel(R) Pentium(R) CPU G4400 @ 3.30GHz (Intel Corporation, Santa Clara, CA, USA)
Memory	3.73 GB
LiDAR	SICK TiM551 (SICK AG, Waldkirch, Germany)
Inertial sensors	LPMS B2 (LP-RESEARCH Inc., Tokyo, Japan)
Wi-Fi 6E network card	Intel WiFi 6E AX210 (Intel Corporation, Santa Clara, CA, USA)
Motor	JSMA000T01_V01 (TECO Electric & Machinery Co., Ltd., Taipei, Taiwan)
Router	ASUS GT-AXE11000 Tri-Band Router (ASUSTeK Computer Inc., Taipei, Taiwan)
Chassis dimensions (length × width × height)	40 cm × 40 cm × 40 cm
Geometry constants (*L*, *H*_1_, *H*_2_)	20 cm, 7.5 cm, 10 cm
LiDAR extrinsic T*ᴮ_L_* (x, y, yaw)	(17.5 cm, 0, 0°)

**Table 4:** End-to-end latency analysis.

No.	Major Processing Block	Main Operations	Straight-Line Test (ms)	90° Turning Test (ms)
1	Data acquisition and synchronization	LiDAR/odometry reception, ROS 2 communication, and timestamp synchronization	17	21
2	LiDAR preprocessing	Coordinate transformation, median filtering, outlier removal, and smoothing	18	24
3	Feature recognition and tracking	AMR feature extraction, ROI update, and linear Kalman filter	18	24
4	Relative angle estimation	KD-tree matching, overlap ratio calculation, and refined dynamic angle search	150	172
5	Virtual-AMR construction	Relative pose fusion and virtual LiDAR generation	12	15
6	AMCL localization update	Virtual scan/TF publication and AMCL update	30	33
Total end-to-end latency			245	289
Equivalent update rate (Hz)			4.08	3.46

**Table 5:** CPU utilization.

Operating Condition	Measured CPU Utilization	Description
Straight-line, refined search	49–66%	Stable ROI and relatively small heading variation
90° turning, refined search	57–71%	More frequent ROI updates and angular search calculations
Initialization/recovery, full-angle search	74–93%	Full angular search dominates computational load

**Table 6:** Sensitivity to the KD-tree correspondence distance gate.

Distance Gate, *ε_d_*	Failure Rate (%)	2D Position RMSE (cm)	Heading RMSE (°)	KD-Tree + Refined-Search Time (ms)	Measured E2E Latency (ms)
1 mm	11.7	5.2	0.38	141	238
2 mm	5.0	4.1	0.24	144	241
3 mm	2.0	3.5	0.16	150	245
4 mm	2.8	3.7	0.18	171	266
5 mm	4.7	4.2	0.24	184	279
7 mm	8.3	5.0	0.34	205	300

**Table 7:** Sensitivity to the angular optimization increment.

Angular Increment, Δ*θ*	Failure Rate (%)	2D Position RMSE (cm)	Heading RMSE (°)	KD-Tree + Refined-Search Time (ms)	Measured E2E Latency (ms)
0.05°	1.5	3.4	0.12	310	405
0.10°	2.0	3.5	0.16	150	245
0.20°	3.3	3.8	0.23	90	185
0.50°	7.5	4.6	0.43	48	143
1.00°	15.0	5.8	0.79	32	127

**Table 8:** AMCL tracking error (unit: m).

AMR	Direction	MAE	RMSE	E_med	E_P95	E_max
A AMR	Y-axis	0.0272	0.0335	0.0225	0.0733	0.1238
	X-axis	0.0323	0.0343	0.0308	0.0781	0.0851
	2D	0.0422	0.0479	0.0437	0.1006	0.1284
B AMR	Y-axis	0.2768	0.3213	0.2406	0.7125	1.6254
	X-axis	0.3294	0.3825	0.2716	0.8473	2.2165
	2D	0.4085	0.4995	0.3919	1.0326	2.7349
Virtual-AMR	Y-axis	0.0249	0.0341	0.0292	0.0846	0.1281
	X-axis	0.0177	0.0256	0.0176	0.0496	0.0796
	2D	0.0336	0.0426	0.0379	0.0949	0.1281

**Table 9:** Particle cloud dispersion statistics (unit: m).

AMR	Direction	MAE	RMSE	E_med	E_P95	E_max
A AMR	Y-axis	0.2049	0.2164	0.1805	0.232	0.2382
	X-axis	0.1907	0.2206	0.1591	0.2284	0.2521
	2D	0.2425	0.3080	0.2449	0.3156	0.3379
B AMR	Y-axis	0.3552	0.3864	0.2641	0.6455	0.6721
	X-axis	0.4093	0.4939	0.3593	0.9781	1.0625
	2D	0.5713	0.6267	0.4517	1.167	1.2556
Virtual-AMR	Y-axis	0.1649	0.1778	0.1577	0.1852	0.1915
	X-axis	0.1846	0.1937	0.1524	0.2175	0.2833
	2D	0.2173	0.2623	0.2138	0.2863	0.3365

**Table 10:** Central-position tracking error (unit: m).

AMR	Direction	MAE	RMSE	E_med	E_P95	E_max
A/B spatial midpoint baseline	Y-axis	0.0336	0.1676	0.0264	0.3487	0.7088
	X-axis	0.1561	0.1754	0.1013	0.4515	1.0209
	2D	0.1585	0.2426	0.2241	0.4765	1.2428
Virtual-AMR	Y-axis	0.0295	0.0479	0.0331	0.0957	0.1307
	X-axis	0.0281	0.0364	0.0347	0.0767	0.1469
	2D	0.0479	0.0597	0.0533	0.1147	0.1486

**Table 11:** Odometry tracking error (unit: m).

AMR	Direction	MAE	RMSE	E_med	E_P95	E_max
A AMR	Y-axis	0.0355	0.0564	0.0479	0.1112	0.1701
	X-axis	0.0496	0.0653	0.0623	0.1656	0.1769
	2D	0.0648	0.0863	0.0902	0.1855	0.2235
B AMR	Y-axis	0.0838	0.1863	0.105	0.386	0.5535
	X-axis	0.0059	0.1621	0.0157	0.3454	0.5492
	2D	0.0891	0.2469	0.1565	0.4023	0.7797
Virtual-AMR	Y-axis	0.0242	0.0305	0.0321	0.0771	0.0863
	X-axis	0.0225	0.0376	0.0294	0.0699	0.1226
	2D	0.0379	0.0475	0.0533	0.0907	0.1275

**Table 12:** Branch-wise ablation configurations.

ID	Configuration	Relative-Position $\left(x,y\right)$ Source	Relative-Heading (*θ*) Source
T1	Standard individual-scan AMCL/direct midpoint baseline	Individual AMCL/TF	Individual AMCL/TF
T2	Relative angle branch only	Individual AMCL/TF	KD-tree angular matching
T3	Feature recognition/tracking branch only	ROI + filtering + Kalman tracking	Individual AMCL/TF
T4	Complete proposed Virtual-AMR method	ROI + filtering + Kalman tracking	KD-tree angular matching

**Table 13:** Straight-line branch-wise ablation error statistics (unit: m).

Test ID	Direction	MAE	RMSE	E_med	E_P95	E_max
T1	Y-axis	0.0336	0.1676	0.0264	0.3487	0.7088
	X-axis	0.1561	0.1754	0.1013	0.4515	1.0209
	2D	0.1585	0.2426	0.2241	0.4765	1.2428
T2	Y-axis	0.0657	0.1371	0.1102	0.2966	0.6217
	X-axis	0.0576	0.0839	0.0475	0.2094	0.5754
	2D	0.0685	0.1607	0.1134	0.3112	0.7364
T3	Y-axis	0.0294	0.0572	0.0425	0.1231	0.2384
	X-axis	0.0713	0.1086	0.0895	0.2006	0.4721
	2D	0.0989	0.1227	0.1101	0.2503	0.5289
T4	Y-axis	0.0249	0.0341	0.0292	0.0846	0.1281
	X-axis	0.0177	0.0256	0.0176	0.0496	0.0796
	2D	0.0336	0.0426	0.0379	0.0949	0.1281

**Table 14:** Turning branch-wise ablation error statistics (unit: m).

Test ID	Direction	MAE	RMSE	E_med	E_P95	E_max
T1	X-axis	0.1949	0.2882	0.0809	0.5092	0.9944
	Y-axis	0.0791	0.1109	0.0711	0.2170	0.4370
	2D	0.2232	0.3088	0.1072	0.5866	1.0594
T2	X-axis	0.1199	0.1730	0.1098	0.3443	0.5643
	Y-axis	0.1030	0.1372	0.0801	0.2740	0.3127
	2D	0.1759	0.2208	0.1503	0.4075	0.6425
T3	X-axis	0.1276	0.1725	0.1320	0.3150	0.4073
	Y-axis	0.1254	0.1684	0.0969	0.3400	0.4399
	2D	0.2023	0.2411	0.1892	0.4027	0.5967
T4	X-axis	0.0292	0.0452	0.0136	0.0821	0.1998
	Y-axis	0.0196	0.0259	0.0154	0.0544	0.1133
	2D	0.0411	0.0521	0.0335	0.0829	0.2297

**Table 15:** Trial-level 2D RMSE across five independent straight-line trials (unit: m).

Test ID	Trial 1	Trial 2	Trial 3	Trial 4	Trial 5	Mean ± SD
T1	0.2201	0.1989	0.2903	0.2638	0.2274	0.2401 ± 0.0365
T2	0.1737	0.1684	0.1771	0.1244	0.1539	0.1595 ± 0.0215
T3	0.1358	0.1256	0.1406	0.1044	0.0986	0.1210 ± 0.0187
T4	0.0299	0.0450	0.0352	0.0503	0.0451	0.0412 ± 0.0083

**Table 16:** Trial-level 2D RMSE across five independent turning trials (unit: m).

Test ID	Trial 1	Trial 2	Trial 3	Trial 4	Trial 5	Mean ± SD
T1	0.2878	0.3143	0.2850	0.3942	0.2422	0.3047 ± 0.0563
T2	0.2024	0.1910	0.2010	0.2127	0.2884	0.2191 ± 0.0395
T3	0.2886	0.2255	0.1843	0.2738	0.2283	0.2401 ± 0.0417
T4	0.0656	0.0538	0.0402	0.0540	0.0419	0.0511 ± 0.0104

## Data Availability

The experimental data supporting the findings of this study are available from the corresponding author upon reasonable request.

## Abbreviations

The following abbreviations are used in this manuscript:

AMCL	Adaptive Monte Carlo localization
AMR	Autonomous mobile robot
DDS	Data Distribution Service
KD-tree	K-dimensional tree
KLD	Kullback–Leibler divergence
MCL	Monte Carlo localization
ROS	Robot operating system
SLAM	Simultaneous localization and mapping
